# Decision, Inference, and Information: Formal Equivalences Under Active Inference

**DOI:** 10.3390/e28010001

**Published:** 2025-12-19

**Authors:** Patrick Sweeney, Jaime Ruiz-Serra, Michael S. Harré

**Affiliations:** Centre for Complex Systems, The University of Sydney, Sydney, NSW 2006, Australia; pswe2854@uni.sydney.edu.au (P.S.); jaime.ruizserra@sydney.edu.au (J.R.-S.)

**Keywords:** active inference, expected free energy, Bayesian decision theory, resource rationality, maximum entropy principle, rate-distortion theory, stochastic optimal control, reinforcement learning, variational inference

## Abstract

A central challenge in artificial intelligence and cognitive science is identifying a unifying principle that governs inference, learning, and action. Active inference proposes such a principle: the minimization of variational free energy. Advocates of active inference argue that the framework subsumes classical models of optimal behavior—including Bayesian decision theory, resource rationality, optimal control, and reinforcement learning—while also instantiating information-theoretic principles such as rate-distortion theory and maximum entropy. However, the literature outlining these conceptual links remains fragmented, limiting integration across fields. This review develops these connections systematically. We show how these major frameworks admit formal correspondences with expected free energy minimization when expressed in variational form, exposing a shared optimization principle that underlies theories of optimal decision-making and information processing. This synthesis is intended both to orient researchers from other fields who are new to active inference and to clarify foundational assumptions for those already working within the framework.

## 1. Introduction

### 1.1. Background and Motivation

Agents operating in uncertain environments must infer hidden states from noisy observations and choose actions with incomplete information. Active inference explains how such agents perceive and act under uncertainty by maintaining and updating a *generative model*: a probabilistic representation of how hidden states cause observations and change over time [1,2,3]. Active inference treats both *perception* and *action* as approximate Bayesian inference. Perception infers hidden states from sensory input. Action selects policies that minimize expected uncertainty and undesired outcomes. Both are driven by a single variational objective: *free energy*, which bounds model evidence and governs belief updates and action selection [4,5,6].

The free energy functional takes two forms, corresponding to the agent’s primary functions: perception and action. In perception, agents minimize *variational free energy* to infer hidden states from observations. In action, agents minimize *expected free energy*, which scores policies based on pragmatic value (achieving goals) and epistemic value (reducing uncertainty) [6]. Both forms of free energy express a single principle: agents perceive and act to minimize surprise.

Active inference is widely promoted as a unifying framework for optimal decision-making under uncertainty, subsuming expected utility theory [3,7], optimal control [8], and reinforcement learning [7,9]. However, accessible, systematic accounts clarifying these connections are scarce. The literature splits into three strands. First, domain-specific surveys tend to address questions within particular disciplinary contexts, offering limited coverage across theoretical domains [1,10,11]. Broader reviews, in turn, primarily emphasize the distinctive properties of active inference, with less attention to its relations with established theoretical traditions [1,12,13,14,15,16]. Third, a prominent body of work treats active inference as a corollary of the free energy principle [17], focusing on the physics of self-organization rather than decision-making under uncertainty [18,19,20,21,22]. Although conceptually rich, this line of work is formally dense and presents a high barrier to entry, making active inference difficult to access for researchers in neighboring fields and limiting integration across disciplines.

Although we do not review statistical physics or the free energy principle in detail, it is worth noting that the functional forms of variational and expected free energy are not arbitrary design choices. Under a path–integral formulation of random dynamical systems interacting with their environment, these functionals emerge as action-like quantities over paths [21]. In this sense, the decision-theoretic special cases reviewed below sit within a broader statistical-physics account of self-organization in open systems, even though our focus here is on their role as objectives for inference and control.

Active inference can be understood as Bayesian decision theory implemented via variational inference. This interpretation clarifies its continuity with existing theories rather than emphasizing superficial differences. This review clarifies active inference’s conceptual foundations and lowers entry barriers by presenting established results in standard notation with minimal technical overhead.

The approach adopted here is significant for understanding techniques in modern artificial intelligence. For instance, the world-class Cicero AI, which outperformed international players in the multi-agent strategy game Diplomacy, used a policy KL-divergence method in its piKL algorithm [23]. This KL-divergence is closely related to the methods discussed in this paper. Understanding the links between algorithms such as piKL and related methods across different research domains will help unify perspectives and advance the broader field.

### 1.2. Objectives and Scope

This review provides a structured reference that situates the expected free energy objective within six established theoretical frameworks, highlighting the relationships between these fields. We group these six frameworks into three perspectives. First, *Bayesian decision theory* and *resource rationality* extend expected utility theory to account for uncertainty and cognitive constraints. Second, *maximum entropy* and *rate-distortion theory* formalize these constraints in terms of the relevant information theory. Third, *stochastic optimal control* and *reinforcement learning* generalize the analysis to sequential settings, integrating inference, planning, and action.

To focus on decision-theoretic structure, we exclude several related topics. First, we consider only discrete-time models. Second, we set aside the broader *free energy principle*, including its continuous-time formulation [21] and the role of the Markov blanket [24,25] in defining system boundaries. Third, we do not address *variational inference* as a model of perception; for foundational treatments, see [5,26,27,28]. Fourth, we omit the *generalized free energy* functional, which jointly optimizes perception and action over time [6]. Fifth, we exclude *novelty-seeking objectives* that reward policies for expected information gain about model parameters, as opposed to salience-seeking objectives pertaining to hidden states [29]. Finally, we exclude *precision updating*, which adjusts action precision according to prediction error [30].

### 1.3. Notation and Conventions

We present active inference in terms of an agent that maintains a *generative model* of its environment, the *generative process* or *world*.

*Probabilities and expectations.* Lowercase letters denote random variables (e.g., o, s, a), and their corresponding probability distributions by the same letters in functional form (e.g., p(o), p(s)). Expectations are written $E_{q(\cdot )}\left[\cdot \right]$, with subscripts indicating the distribution of integration. For example, $E_{q(o|a)}\left[\ln p\left(o|a\right)\right]$ denotes the expectation of lnp(o∣a) when *o* is drawn from the conditional distribution q(o∣a), with *a* treated as fixed. Throughout, all probability distributions are assumed to be *discrete*, corresponding to categorical random variables with Dirichlet priors representing uncertainty over their parameters.

*Generative process.* We formalize the environment as a *partially observable Markov decision process* (POMDP) [31]. At each time step *t*, the environment occupies a hidden state $w_{t}\in W$, emits an observation $o_{t}\in O$, and receives an action $a_{t}\in A$ from the agent. The trajectory of environment states, actions, and observations is denoted by $w_{1:T},\;a_{1:T},\;o_{1:T}$.

*Generative model.* The agent maintains a probabilistic generative model over hidden states $s_{t}\in S$, observations $o_{t}\in O$, and actions $a_{t}\in A$. At each time step *t*, it receives an observation $o_{t}$, updates its belief over the hidden state $s_{t}$ given past observations, and then evaluates possible actions $a_{t}$ under its model. The agent’s internal trajectories are denoted by $s_{1:T},\;a_{1:T},\;o_{1:T}$. We assume a Markov policy that conditions on the current hidden state, $\pi (a_{t}|s_{t})$. Given this policy, the joint distribution over trajectories induced by the generative model and the policy factorizes as

(1) $$p\left(s_{1:T},\;o_{1:T},\;a_{1:T}\right)=p\left(s_{1}\right)\left[\prod_{t=1}^{T}p\left(o_{t}|s_{t}\right)\;\pi \left(a_{t}|s_{t}\right)\right]\left[\prod_{t=1}^{T-1}p\left(s_{t+1}|s_{t},\;a_{t}\right)\right]$$

where $p(s_{1})$ is the *prior* over states, $p(o_{t}|s_{t})$ is the *observation model*, $p(s_{t+1}|s_{t},\;a_{t})$ is the *state transition model*, and $\pi (a_{t}|s_{t})$ is the *policy* mapping the current state to a distribution over actions. This induces a dynamic Bayesian network [32] in which, conditional on $s_{t}$, the current observation and action are independent of the past, and conditional on $\left(s_{t},\;a_{t}\right)$ the next state $s_{t+1}$ is independent of earlier variables. When the dependence on $s_{t}$ is clear from context, we write $\pi (a_{t})$ for brevity. The agent’s goals are encoded in a separate preference distribution over observations, denoted by $p^{*}\left(o_{t}\right)$.

*Variational beliefs.* The agent uses variational inference to approximate otherwise intractable posteriors with a restricted family (e.g., mean-field or structured parameterizations), so that prediction and evaluation of candidate actions reduce to a tractable optimization problem [2,5]. Its generative model is denoted by p(·), and its approximate posterior by q(·). Given past observations $o_{1:t}$, the exact filtering distribution $p(s_{t}|o_{1:t})$ [33] is approximated by a variational distribution $q(s_{t}|o_{1:t})$; for notational economy, we write $q(s_{t})$ when the conditioning on $o_{1:t}$ is clear from context. For a fixed candidate action $a_{t}$, the agent forms one-step-ahead predictive beliefs by marginalizing over the current state:

(2) $$\begin{array}{cc} q(s_{t+1}|a_{t},\;o_{1:t}) & =\sum_{s_{t}}p\left(s_{t+1}|s_{t},\;a_{t}\right)\;q\left(s_{t}|o_{1:t}\right) \end{array}$$

(3) $$\begin{array}{cc} q(o_{t+1}|a_{t},\;o_{1:t}) & =\sum_{s_{t+1}}p\left(o_{t+1}|s_{t+1}\right)\;q\left(s_{t+1}|a_{t},\;o_{1:t}\right) \end{array}$$

where we assume that, conditional on the next hidden state, the observation likelihood does not depend on the chosen action, i.e., $p\left(o_{t+1}|s_{t+1},\;a_{t}\right)=p\left(o_{t+1}|s_{t+1}\right)$ for all $a_{t}$.

*Actions and policies.* Our terminology departs from typical conventions in the active inference literature in two respects. First, we use the term *action* to refer to what the active inference literature calls *control states*: counterfactual interventions considered during planning. Since our treatment is limited to this planning context, we do not distinguish between control states and executed actions. Second, what the active inference literature calls a *policy*—a sequence of candidate actions—we call a *plan*. A plan is an action sequence $a_{1:T}=\left(a_{1},\;a_{2},\ldots ,a_{T}\right)$. For a one-step horizon (T=1), we write the candidate action as $a=a_{1}$. We reserve the term *policy* for any probability distribution over actions or plans. Thus, π(a) denotes a policy distribution over actions, and $\pi (a_{1:T})$ a policy distribution over plans. In reinforcement learning and stochastic control, a policy typically maps each state s∈S to a distribution over actions, π(a∣s). We preserve this definition when referencing those frameworks.

*Expected free energy.* We distinguish two levels at which expected free energy is evaluated: over individual actions and over distributions of actions (policies). The *expected free energy of an action*, G(a), quantifies the expected outcome under current beliefs given a specific candidate action. The *expected free energy of a policy*, G[π], evaluates the expected free energy under a distribution over actions π(a).

*Time index conventions.* We streamline notation by suppressing explicit time indices except when required for dynamic programming. To represent one-step prediction, we use primed variables: $s^{\prime }∼q\left(s^{\prime }|s,\;a\right)$ denotes the transition model otherwise written as $q(s_{t+1}|s_{t},\;a_{t})$, and $o^{\prime }∼q\left(o^{\prime }|s^{\prime }\right)$ denotes the observation model $q(o_{t+1}|s_{t+1})$.

*Functionals and information-theoretic quantities.* We use parentheses for functions and square brackets for functionals: L[·] denotes a Lagrangian, H[q] entropy, H[q, p] cross-entropy, $D_{\mathrm{KL}}\left[q\;||\;p\right]$ Kullback-Leibler (KL) divergence, and I[q;p] mutual information. All logarithms are natural and information is measured in nats.

## 2. Expected Free Energy

Active inference assumes that agents choose actions (or multi-step plans) that minimize an *expected free energy* functional [2,13,34]. This is a modeling postulate about the agent’s decision rule, analogous to the assumption in Bayesian decision theory that agents maximize expected utility. The functional is defined so that low values correspond to actions that (i) make preferred observations likely and (ii) are expected to reduce future uncertainty about hidden states. In this way, expected free energy combines two basic drives in a single scalar objective: achieving preferred outcomes (exploitation) and improving the generative model through information gain (exploration) [35,36].

In the one-step setting considered here, given a candidate action *a*, the expected free energy G(a) is defined as a functional of the joint *posterior predictive distribution*$q(o^{\prime },\;s^{\prime }|a)$ of future observations $o^{\prime }$ and states $s^{\prime }$:

(4) $$G\left(a\right)=E_{q(o^{\prime },\;s^{\prime }|a)}\left[\ln q\left(o^{\prime }|a\right)-\ln p^{*}\left(o^{\prime }\right)-\ln p\left(o^{\prime }|s^{\prime }\right)\right]$$

Here $o^{\prime }$ denotes a *future* observation, treated as a random variable under $q(o^{\prime },\;s^{\prime }|a)$, in contrast to the realized past observations $o_{1:t}$ that appear as fixed conditioning arguments in $p(s_{t}|o_{1:t})$.

The agent’s *preference distribution*, $p^{*}\left(o\right)$, defines a fixed, exogenous preference over observations that encode its goals. This distribution assigns high probability to ‘desirable’ outcomes that are characteristic of the system or agent in question (i.e., the outcomes that are characteristic of the kind of system it is, such that adaptive systems are just those systems that are highly unlikely to be in uncharacteristic states) [2].

Using the linearity of expectation, we separate the terms of G(a):

(5) $$G\left(a\right)=E_{q(o^{\prime },s^{\prime }|a)}\left[\ln q\left(o^{\prime }|a\right)\right]-E_{q(o^{\prime },s^{\prime }|a)}\left[\ln p^{*}\left(o^{\prime }\right)\right]-E_{q(o^{\prime },s^{\prime }|a)}\left[\ln p\left(o^{\prime }|s^{\prime }\right)\right]$$

The first two terms depend only on $o^{\prime }$, so we rewrite them as expectations under $q(o^{\prime }\;|\;a)$. The third term depends on both $s^{\prime }$ and $o^{\prime }$, which we rewrite using the law of total expectation:

(6) $$G\left(a\right)=\underset{\mathrm{Cross}-\mathrm{entropy}}{\underset{︸}{-E_{q(o^{\prime }|a)}\left[\ln p^{*}\left(o^{\prime }\right)\right]}}-\underset{\mathrm{Entropy}}{\underset{︸}{\left(-E_{q(o^{\prime }|a)}\left[\ln q\left(o^{\prime }|a\right)\right]\right)}}+\underset{\mathrm{Conditional}\;\mathrm{entropy}}{\underset{︸}{\left(-E_{q(s^{\prime }|a)}\left[E_{q(o^{\prime }|s^{\prime },a)}\left[\ln p\left(o^{\prime }|s^{\prime }\right)\right]\right]\right)}}$$

The generative model defines the observation likelihood, so we substitute $q\left(o^{\prime }|s^{\prime },\;a\right):=p\left(o^{\prime }|s^{\prime }\right)$ to simplify the conditional entropy:

(7) $$G\left(a\right)=\underset{\mathrm{Cross}-\mathrm{entropy}}{\underset{︸}{-E_{q(o^{\prime }|a)}\left[\ln p^{*}\left(o^{\prime }\right)\right]}}-\underset{\mathrm{Entropy}}{\underset{︸}{\left(-E_{q(o^{\prime }|a)}\left[\ln q\left(o^{\prime }|a\right)\right]\right)}}+\underset{\mathrm{Conditional}\;\mathrm{entropy}}{\underset{︸}{E_{q(s^{\prime }|a)}\left[H[p\left(o^{\prime }|s^{\prime }\right)]\right]}}$$

By regrouping the entropy term with either the cross-entropy or the conditional entropy, we recover two equivalent decompositions of expected free energy, each associated with a familiar interpretation in the literature.

### 2.1. Pragmatic and Epistemic Value

The first decomposition separates expected free energy into two terms: *pragmatic value* and *epistemic value*.

(8) $$G\left(a\right)=-\underset{\mathrm{Pragmatic}\;\mathrm{value}}{\underset{︸}{E_{q(o^{\prime }|a)}\;\left[\ln p^{*}\left(o^{\prime }\right)\right]}}-\underset{\mathrm{Epistemic}\;\mathrm{value}}{\underset{︸}{E_{q(s^{\prime },o^{\prime }|a)}\left[\ln q\left(o^{\prime }|s^{\prime },a\right)-\ln q\left(o^{\prime }|a\right)\right]}}$$

Pragmatic value reflects how closely the predicted observations $q(o^{\prime }|a)$ match the agent’s preferences $p^{*}\left(o^{\prime }\right)$. Formally, it equals the negative cross-entropy $-H(q,\;p^{*})$, which reaches its maximum when the agent expects to realize the observations it prefers most.

Epistemic value, or *salience*, is the conditional mutual information between future states ($s^{\prime }$) and observations ($o^{\prime }$), given the action [37]. It measures how much the agent expects to reduce uncertainty about hidden states by choosing actions that produce informative observations that help distinguish between competing hypotheses.

(9) $$G\left(a\right)=-\underset{\mathrm{Pragmatic}\;\mathrm{value}}{\underset{︸}{E_{q(o^{\prime }|a)}\;\left[\ln p^{*}\left(o^{\prime }\right)\right]}}-\underset{\mathrm{Epistemic}\;\mathrm{value}}{\underset{︸}{I_{q(s^{\prime },o^{\prime }|a)}\left[s^{\prime };o^{\prime }|a\right]}}$$

Equivalently, epistemic value measures expected *information gain*, defined as the expected KL divergence between the agent’s prior and posterior state estimates:

(10) $$G\left(a\right)=-\underset{\mathrm{Pragmatic}\;\mathrm{value}}{\underset{︸}{E_{q(o^{\prime }|a)}\;\left[\ln p^{*}\left(o^{\prime }\right)\right]}}-\underset{\mathrm{Epistemic}\;\mathrm{value}}{\underset{︸}{E_{q(o^{\prime }|a)}\;\left[D_{\mathrm{KL}}\left[q\left(s^{\prime }|o^{\prime },a\right)\;∥\;q\left(s^{\prime }|a\right)\right]\right]}}$$

A large information gain means the agent expects to update its beliefs substantially after observing $o^{\prime }$, and therefore sees the observation as highly informative. Section 3 develops this connection between epistemic value and information gain.

### 2.2. Risk and Ambiguity

Alternatively, expected free energy decomposes into two aversive components: *risk* and *ambiguity*.

(11) $$G\left(a\right)=\underset{\mathrm{Risk}}{\underset{︸}{D_{\mathrm{KL}}\;\left[q\left(o^{\prime }|a\right)\;∥\;p^{*}\left(o^{\prime }\right)\right]}}+\underset{\mathrm{Ambiguity}}{\underset{︸}{E_{q(s^{\prime }|a)}H\left[p\left(o^{\prime }|s^{\prime }\right)\right]}}$$

Risk measures the KL divergence between predicted and preferred outcomes. It falls to zero when predictions match preferences exactly, and rises as the mismatch grows.

Ambiguity captures the expected uncertainty in the observation model, given the predictive state distribution. Formally, it is the expected conditional entropy: $E_{p(s^{\prime })}\left[H\left[p\left(o^{\prime }|s^{\prime }\right)\right]\right]$, where $p(o^{\prime }|s^{\prime })$ is the observation model and $p(s^{\prime })$ is the predictive state distribution. High ambiguity means that observations depend only weakly on hidden states and provide little information. Low ambiguity means that observations reliably reveal hidden causes.

To show that Equation (11) is a reexpression of Equation (9), we expand the KL divergence:

(12) $$G\left(a\right)=\underset{\mathrm{Pragmatic}\;\mathrm{value}}{\underset{︸}{-E_{q(o^{\prime }|a)}\left[\ln p^{*}\left(o^{\prime }\right)\right]}}-\underset{\mathrm{Entropy}}{\underset{︸}{H[q\left(o^{\prime }|a\right)]}}+\underset{\mathrm{Ambiguity}}{\underset{︸}{E_{q(s^{\prime }|a)}H\left[p\left(o^{\prime }|s^{\prime }\right)\right]}}$$

The sum of entropy and ambiguity equals the epistemic value:

(13) $$G\left(a\right)=-\underset{\mathrm{Pragmatic}\;\mathrm{value}}{\underset{︸}{E_{q(o^{\prime }|a)}\left[\ln p^{*}\left(o^{\prime }\right)\right]}}-(\underset{\mathrm{Entropy}}{\underset{︸}{H[q\left(o^{\prime }|a\right)]}}-\underset{\mathrm{Ambiguity}}{\underset{︸}{E_{q(s^{\prime }|a)}H\left[p\left(o^{\prime }|s^{\prime }\right)\right]}})$$

The epistemic value equals the mutual information:

(14) $$\underset{\mathrm{Epistemic}\;\mathrm{value}}{\underset{︸}{I_{q(s^{\prime },o^{\prime }|a)}\left[s^{\prime };\;o^{\prime }|a\right]}}=\underset{\mathrm{Entropy}}{\underset{︸}{H[q\left(o^{\prime }|a\right)]}}-\underset{\mathrm{Ambiguity}}{\underset{︸}{E_{q(s^{\prime }|a)}H\left[p\left(o^{\prime }|s^{\prime }\right)\right]}}$$

Substituting the mutual information of Equation (14) into Equation (13) recovers the pragmatic–epistemic decomposition in Equation (9).

### 2.3. Optimal Policy

Agents select *policies* by minimizing expected free energy, which trades off expected cost against consistency with *prior habits*. The expected free energy of a policy G[π] is defined as follows:

(15) $$G\left[\pi \right]=\sum_{a}\pi \left(a\right)\;G\left(a\right)+\frac{1}{\gamma }D_{\mathrm{KL}}\left[\pi \left(a\right)\;∥\;\pi_{0}\left(a\right)\right]$$

Here, π(a) denotes the posterior policy, G(a) the expected free energy associated with action *a*, and $\pi_{0}\left(a\right)$ a prior policy representing habitual tendencies in the absence of deliberation [38]. The KL divergence term measures how much the posterior policy π(a) deviates from the prior $\pi_{0}\left(a\right)$, while the precision parameter γ controls the strength of this deviation penalty. In this formulation, we treat $\pi_{0}\left(a\right)$ as fixed, though in general it can itself be learned.

Minimizing G[π] yields the *optimal posterior policy* $\pi^{*}\left(a\right)$:

(16) $$\pi^{*}\left(a\right)=\frac{\pi_{0}\left(a\right)\exp\left[-\gamma \;G\left(a\right)\right]}{\sum_{a^{\prime }}\pi_{0}\left(a^{\prime }\right)\exp\left[-\gamma \;G\left(a^{\prime }\right)\right]}$$

This takes the canonical softmax form:

(17) $$\pi^{*}\left(a\right)=\sigma_{a}\;\left(\ln\pi_{0}\left(a\right)-\gamma \;G\left(a\right)\right)$$

where $\sigma_{a}\left(\cdot \right)$ denotes the softmax operator over actions.

The optimal policy favors actions with low expected free energy while staying close to habitual preferences encoded by the prior. The precision parameter γ determines their relative influence: high γ makes the policy more selective for actions with low G(a), whereas low γ increases the weight of prior habits [39].

### 2.4. Planning as Inference

This framework generalizes from single actions to full sequences of actions, or *plans*, $a_{1:T}=\left(a_{1},\ldots ,a_{T}\right)$. Preferences are now defined over sequences of future observations, represented by $p^{*}\left(o_{1:T}\right)$.

The expected free energy of a plan is the sum of stepwise contributions, each depending on the preceding actions through their influence on predicted states and outcomes:

(18) $$G_{T}\left(a_{1:T}\right)=\sum_{t=1}^{T}G_{t}\left(a_{t}|a_{1:t-1}\right)$$

The sum in Equation (18) represents the accumulation of expected free energy along the trajectory. In this sense, $G_{T}$ plays the role of an *action functional* over paths $a_{1:T}$: plans with low expected free energy correspond to ’least-action’ paths under the agent’s generative model and preference distribution [21,40].

Given a planning horizon *T*, the agent infers the optimal posterior policy $\pi_{T}^{*}\left(a_{1:T}\right)$ over full sequences:

(19) $$\pi_{T}^{*}\left(a_{1:T}\right)=\frac{\pi_{0}\left(a_{1:T}\right)\;\exp\left[-\gamma G_{T}\left(a_{1:T}\right)\right]}{\sum_{a_{1:T}^{\prime }}\pi_{0}\left(a_{1:T}^{\prime }\right)\;\exp\left[-\gamma G_{T}\left(a_{1:T}^{\prime }\right)\right]}$$

This posterior favors action sequences with low expected free energy, weighted by their prior probability under $\pi_{0}\left(a_{1:T}\right)$. If the prior over action sequences is uniform, the posterior simplifies to a standard softmax over $-\;G_{T}\left(a_{1:T}\right)$, so that plans with lower expected free energy receive higher probability.

### 2.5. A Common Decomposition of Objectives

Expected free energy provides a compact way to decompose and compare the objectives used in several decision-theoretic and control frameworks. Equation (7) gathers terms that already appear, in different combinations, across these approaches into a single expression. Each framework can be written so that its objective retains a subset of these components, yielding different optimization criteria, which differ in which terms they emphasize (pragmatic value, entropy, or ambiguity) and in whether they induce deterministic argmax policies or “soft” stochastic policies via the precision-weighting mechanism described in Section 2.3. Table 1 summarizes these relationships.

## 3. Bayesian Decision Theory

Bayesian decision theory defines the standard of rational behavior in artificial intelligence, describing how an ideal agent acts optimally under uncertainty [36]. An agent following this theory selects actions that maximize *expected utility*, given a subjective probability model over hidden states and observations [41,42]. Active inference extends this framework by incorporating *Bayesian optimal design* [43,44,45], enabling agents to maximize both *instrumental utility* [46] and the *expected utility of information gain* [37]. Both terms rely on the same logarithmic scoring rule, which expresses utility and information gain in the same units: nats. This shared scale permits the agent to combine both quantities into a single minimization objective: the expected free energy.

### 3.1. Pragmatic Value as Expected Utility

Active inference departs from classical decision theory by representing preferences as probability distributions rather than scalar utilities [47]. Instead of assigning real-valued scores to outcomes, agents encode desired observations as a preference distribution $p^{*}\left(o\right)$. We can recover a real-valued utility function by taking this distribution’s logarithm [48,49]:

(20) $$u\left(o\right):=\ln p^{*}\left(o\right)$$

Because $p^{*}\left(o\right)\in \left(0,1\right]$, utilities lie in (−∞, 0]: highly preferred outcome approach 0, and less preferred outcomes approach negative infinity.

Given predicted observations $q(o^{\prime }|a)$, the agent’s expected utility equals its pragmatic value (Equation (8)):

(21) $$\underset{\mathrm{Expected}\;\mathrm{utility}}{\underset{︸}{E_{q(o^{\prime }|a)}\left[u\left(o^{\prime }\right)\right]}}=\underset{\mathrm{Pragmatic}\;\mathrm{value}}{\underset{︸}{E_{q(o^{\prime }|a)}\left[\ln p^{*}\left(o^{\prime }\right)\right]}}$$

There are two complementary interpretations of this identity: one from the perspective of proper scoring rules and one from information theory.

First, from the viewpoint of proper scoring rules, the logarithmic utility $u\left(o\right)=\ln p^{*}\left(o\right)$ corresponds to the *logarithmic score*, which is the unique strictly proper scoring rule that rewards accurate probabilistic forecasting [50,51].

(22) $$\underset{\mathrm{Pragmatic}\;\mathrm{value}}{\underset{︸}{E_{q(o^{\prime }|a)}\left[\ln p^{*}\left(o^{\prime }\right)\right]}}=\underset{\mathrm{Expected}\;\mathrm{proper}\;\mathrm{score}}{\underset{︸}{E_{q(o^{\prime }|a)}\left[S\left(p^{*},o^{\prime }\right)\right]}}$$

Under this interpretation, the world acts as the *scorer*, evaluating the agent’s preferences against the distribution it actually generates. Expected utility is the *expected score* that the agent earns for its preferences $p^{*}$ when outcomes are drawn from *q*. When $p^{*}=q$, the agent achieves the maximal expected score, reflecting perfect calibration between preference and reality. This property generalizes within the theory of proper scoring rules, where each rule defines a convex potential on the space of probability distributions and induces an associated information geometry [52,53,54,55]. The detailed development of this connection lies beyond the present scope.

Second, from the viewpoint of information theory, the same identity reveals the informational limits of utility. Expected utility equals the negative cross-entropy between predicted outcomes $q(o^{\prime }|a)$ and preferred outcomes $p^{*}\left(o^{\prime }\right)$:

(23) $$\underset{\mathrm{Pragmatic}\;\mathrm{value}}{\underset{︸}{E_{q(o^{\prime }|a)}\left[\ln p^{*}\left(o^{\prime }\right)\right]}}=\underset{\mathrm{Negative}\;\mathrm{cross}-\mathrm{entropy}}{\underset{︸}{-H[q,p^{*}]}}$$

Cross-entropy measures the expected number of nats required to encode outcomes from *q* using a code optimized for $p^{*}$ [56]. Maximizing expected utility reduces this coding cost and aligns predicted with preferred outcomes.

Expanding the cross-entropy, $H\left[q,\;p^{*}\right]=H\left[q\right]+D_{\mathrm{KL}}\left[q\;∥\;p^{*}\right]$, gives the canonical decomposition:

(24) $$\underset{\mathrm{Expected}\;\mathrm{utility}}{\underset{︸}{E_{q(o^{\prime }|a)}\left[u\left(o^{\prime }\right)\right]}}=\underset{\mathrm{Negative}\;\mathrm{risk}}{\underset{︸}{-D_{\mathrm{KL}}\;\left[q\left(o^{\prime }|a\right)\;∥\;p^{*}\left(o^{\prime }\right)\right]}}-\underset{\mathrm{Entropy}\;\mathrm{of}\;\mathrm{predicted}\;\mathrm{outcomes}}{\underset{︸}{H\;\left[q(o^{\prime }|a)\right]}}.$$

This decomposition shows that expected utility is bounded above by the negative entropy of the world:

(25) $$E_{q}\left[u\left(o^{\prime }\right)\right]\leq -H\left[q\right]$$

with equality when $q=p^{*}$. Entropy sets the fundamental informational ceiling on achievable utility. The KL term measures self-inflicted loss, the information cost of preference–prediction misalignment, while the entropy term captures the irreducible uncertainty of the environment.

Together, these two interpretations highlight different aspects of the same structure: proper scoring rules describe the normative geometry of truthful probabilistic reporting, while information theory describes the limits of achievable utility. Both converge on the same principle: utility and accuracy are governed by entropy and divergence in the shared geometry of distributions [53,57].

### 3.2. Salience as the Value of Information

In active inference, *salience* or *epistemic value* is the expected information gain about hidden states. This parallels *Bayesian optimal design*, where experiments are chosen to maximize expected information utility [43,44]. Under active inference, this expected utility is again expressed through the logarithmic scoring rule.

The logarithmic score u(w)=lnq(w) typically evaluates how well a probabilistic forecast *q* predicts an observed world state *w*. Because this world state is hidden, the agent instead applies the same rule *internally*: u(s)=lnq(s). Its expected value, $E_{q(s)}\left[\ln q\left(s\right)\right]=-H\left[q\left(s\right)\right]$, quantifies the certainty of the agent’s belief state. Information gain is the expected increase in this self-score that occurs when the agent acts, observes the outcome, and updates its beliefs [53,58,59]. Note that this utility is entirely distinct from the utility function in Equation (21). Here, the utility reflects the value of uncertainty reduction for its own sake, rather than an expression of instrumental or goal-directed preference.

After taking action *a* and transitioning to a new hidden state, the agent forms a posterior predictive belief $q(s^{\prime }|a)$ over hidden states $s^{\prime }$, and scores this belief using $u\left(s^{\prime }\right):=\ln q\left(s^{\prime }|a\right)$. The expected utility is the expected log score under *q*, which equals the negative entropy:

(26) $$\underset{\begin{array}{c} \mathrm{Expected}\;\mathrm{utility}\\ \left(\mathrm{prior}\right) \end{array}}{\underset{︸}{E_{q}\left[u|a\right]}}=\underset{\begin{array}{c} \mathrm{Expected}\;\log\;\mathrm{score}\\ \left(\mathrm{prior}\right) \end{array}}{\underset{︸}{E_{q(s^{\prime }|a)}\left[\ln q\left(s^{\prime }|a\right)\right]}}=\underset{\begin{array}{c} \mathrm{Negative}\;\mathrm{entropy}\\ \left(\mathrm{prior}\right) \end{array}}{\underset{︸}{-H[q\left(s^{\prime }|a\right)]}}$$

After observing $o^{\prime }$, the agent updates its belief to $q(s^{\prime }|o^{\prime },\;a)$. It then evaluates the new, more informed belief using the same scoring rule. Its expected utility becomes the expected negative entropy of the posterior:

(27) $$\underset{\begin{array}{c} \mathrm{Expected}\;\mathrm{utility}\\ \left(\mathrm{posterior}\right) \end{array}}{\underset{︸}{E_{q}\left[u|a,\;o^{\prime }\right]}}=\underset{\begin{array}{c} \mathrm{Expected}\;\log\;\mathrm{score}\\ \left(\mathrm{posterior}\right) \end{array}}{\underset{︸}{E_{q(o^{\prime }|a)}\left[E_{q(s^{\prime }|o^{\prime },\;a)}\left[\ln q\left(s^{\prime }|o^{\prime },a\right)\right]\right]}}=\underset{\begin{array}{c} \mathrm{Negative}\;\mathrm{entropy}\\ \left(\mathrm{posterior}\right) \end{array}}{\underset{︸}{-E_{q(o^{\prime }|a)}\left[H[q\left(s^{\prime }|o^{\prime },\;a\right)]\right]}}$$

With the information inequality [60], conditioning cannot increase entropy on average: the expected posterior uncertainty is never greater than the prior uncertainty. Thus, the agent anticipates that updating on new evidence will make its beliefs at least as precise as before. The *expected value of sample information* (EVSI) [61] measures this expected improvement in utility:

(28) $$\begin{array}{cc} \mathrm{EVSI}(a)\; & =\underset{\begin{array}{c} \mathrm{Expected}\;\mathrm{utility}\\ \left(\mathrm{posterior}\right) \end{array}}{\underset{︸}{E_{q}\left[u|a,\;o^{\prime }\right]}}-\underset{\begin{array}{c} \mathrm{Expected}\;\mathrm{utility}\\ \left(\mathrm{prior}\right) \end{array}}{\underset{︸}{E_{q}\left[u|a\right]}}\\ \; & =\underset{\begin{array}{c} \mathrm{Negative}\;\mathrm{entropy}\\ \left(\mathrm{posterior}\right) \end{array}}{\underset{︸}{-E_{q(o^{\prime }|a)}\left[-H[q\left(s^{\prime }|o^{\prime },\;a\right)]\right]}}-\underset{\begin{array}{c} \mathrm{Negative}\;\mathrm{entropy}\\ \left(\mathrm{prior}\right) \end{array}}{\underset{︸}{-H[q\left(s^{\prime }|a\right)]}}\geq 0 \end{array}$$

This expression equals the conditional mutual information between $s^{\prime }$ and $o^{\prime }$ given *a*. It also corresponds to the expected information gain:

(29) $$\mathrm{EVSI}\left(a\right)=\underset{\mathrm{Conditional}\;\mathrm{mutual}\;\mathrm{information}}{\underset{︸}{I_{q(s^{\prime },o^{\prime }|a)}\left[s^{\prime };o^{\prime }|a\right]}}=\underset{\mathrm{Expected}\;\mathrm{information}\;\mathrm{gain}}{\underset{︸}{E_{q(o^{\prime }|a)}\left[D_{\mathrm{KL}}\;\left[q\left(s^{\prime }|o^{\prime },\;a\right)\;∥\;q\left(s^{\prime }|a\right)\right]\right]}}$$

### 3.3. Intrinsic Value and Curiosity

Curiosity, understood as a drive to resolve uncertainty, has strong roots in neuroscience and decision theory [62,63]. Empirical studies show that the brain encodes uncertainty to guide active sampling, and that this process shapes attention, learning, and goal-directed behavior [64,65]. These findings have inspired artificial agents that learn through intrinsic motivation [66,67]. Early approaches used information-theoretic principles to drive exploration. These included entropy-based experiment design [68], active learning with uncertainty-aware models [69], and exploratory reinforcement learning through information gain [70]. Later, Bayesian strategies helped agents manage the trade-off between exploration and exploitation [71].

Researchers subsequently formalized curiosity as the drive to maximize expected information gain. They used mutual information and entropy-based objectives to model Bayesian surprise [72], optimize variational information gain [73], and track learning progress under epistemic uncertainty [74]. Mutual information between states and actions has guided unsupervised skill acquisition [75], while diversity-based objectives have enabled agents to discover skills without external rewards [76]. Recent work has combined these ideas into broader frameworks: self-supervised world modeling [77], meta-learning via variational Bayes [78], reinforcement learning with information–regret trade-offs [79,80], and unified objectives that directly optimize information gain [81,82].

## 4. Resource-Rational Decision Theory

The expected utility framework assumes agents are perfectly rational, with unlimited computational capacity and access to complete information, allowing them to maximize subjective expected utility across all possible outcomes [41]. However, this idealization neglects the computational and informational limits of real cognitive systems. Recent research in the cognitive sciences increasingly supports the view that intelligent behavior reflects utility maximization under resource constraints [83,84,85,86,87,88]. This view extends Simon’s [89] notion of *bounded rationality*, which frames rationality as limited by incomplete information, finite cognitive capacity, and the constraints of time [90].

Formal accounts model resource rationality as constrained optimization: agents trade off expected utility against information-theoretic costs, such as the effort of deliberation or deviation from default policies [91,92,93,94,95]. These trade-offs yield soft policies with stochastic action selection. Active inference can be written in the same variational form as these approaches, with expected free energy playing the role of the cost.

### 4.1. Value–Information Trade-Off

We consider an agent that selects a stochastic policy π, where π(a) gives the probability of choosing action *a*. The agent aims to minimize the expected cost $E_{a∼\pi }\left[G\left(a\right)\right]$, subject to a constraint *K* on the divergence from a prior policy $\pi_{0}$.

(30) $$\begin{array}{cc} \min_{\pi (a)}\; & E_{\pi (a)}\left[G\left(a\right)\right]\\ \mathrm{subject}\;\mathrm{to}\; & D_{\mathrm{KL}}\left[\pi \left(a\right)\;∥\;\pi_{0}\left(a\right)\right]\leq K \end{array}$$

Equivalently, the agent maximizes the sum of expected utility and expected information gain, as shown in Equation (8), under the same constraint.

To solve this constrained optimization problem, we introduce the Lagrangian:

(31) $$L\left[\pi ,\;\gamma ,\;\lambda \right]=\sum_{a}\pi \left(a\right)G\left(a\right)+\frac{1}{\gamma }\left(D_{\mathrm{KL}}\left[\pi \left(a\right)\;∥\;\pi_{0}\left(a\right)\right]-K\right)+\lambda \left(\sum_{a}\pi \left(a\right)-1\right)$$

Differentiating L with respect to π(a) and setting the result to zero gives the stationarity condition:

$$\begin{array}{cc} \frac{\partial L}{\partial \pi (a)}\; & =G\left(a\right)+\frac{1}{\gamma }\left(\ln\frac{\pi (a)}{\pi_{0}\left(a\right)}+1\right)+\lambda =0\\ \Rightarrow \;\ln\frac{\pi (a)}{\pi_{0}\left(a\right)}\; & \propto -\gamma G(a)\\ \Rightarrow \;\pi^{*}\left(a\right)\; & \propto \pi_{0}\left(a\right)\exp\left[-\gamma G(a)\right] \end{array}$$

Normalising yields the optimal posterior policy defined in Equation (16).

### 4.2. Metareasoning

Neural systems allocate cognitive effort adaptively, relying on mechanisms for sampling, cost monitoring, and arbitration between fast and deliberative control processes [96,97,98,99,100]. We can formalize this adaptive process as *metareasoning*: deciding how much computation to perform before acting [101,102,103,104]. Early models of metareasoning treated agents as ideal reasoners, capable of unlimited deliberation about their own decision processes. But this leads to an infinite regress: each decision about what to compute next requires further computation to evaluate its value.

Ortega and Braun [92] avoid this problem by framing metareasoning as a sequential decision problem under uncertainty. In their model, internal computation is treated as bounded evidence accumulation. The agent plays a Bayesian game against Nature, who privately determines the cost of continued deliberation and may halt the process at any time. This setup highlights a central trade-off: more computation can improve decisions, but it also incurs increasing costs. To evaluate this trade-off, the agent needs a common currency to compare computational costs with expected utility gains.

Active inference provides this currency by expressing both information-processing costs and utility gains in the same units: nats, the natural unit of information. The precision parameter γ acts as an exchange rate, converting utility into information. This enables trade-offs between expected reward and computational cost, and parallels the rational inattention framework in economics, where the precision parameter γ functions as a shadow price on attention or information acquisition [105,106,107].

## 5. Maximum Entropy Principle

Active inference implements the Maximum Entropy Principle (MaxEnt) by maximizing policy entropy subject to constraints [20]. According to MaxEnt, the probability distribution that maximizes entropy subject to known constraints best represents the current state of knowledge, implementing Occam’s razor by choosing the least biased distribution consistent with the available information [108,109,110,111]. This principle can be justified axiomatically as the only inference rule consistent with rationality, invariance, and independence [112]. More generally, MaxEnt is a special case of the *minimum divergence principle* [113,114,115], which identifies the distribution minimizing KL divergence to a reference prior under moment constraints. When this prior is uniform, the result coincides with the maximum entropy distribution. Beyond its role as an inference principle, MaxEnt has also been used to motivate principles of minimum entropy production [116] and to analyze dissipative structures in non-equilibrium thermodynamics [117], connections we do not pursue here.

### 5.1. Minimum Divergence Principle

Active inference instantiates the minimum divergence principle: the agent chooses a policy π(a) that stays as close as possible to a reference policy $\pi_{0}\left(a\right)$, while keeping expected free energy below a fixed bound *C* [118,119]. This yields the following constrained optimization problem:

(32) $$\begin{array}{cc} \min_{\pi (a)}\; & D_{\mathrm{KL}}\left[\pi \left(a\right)\;∥\;\pi_{0}\left(a\right)\right]\\ \mathrm{subject}\;\mathrm{to}\; & E_{\pi (a)}\left[G\left(a\right)\right]\leq C \end{array}$$

To solve this, we introduce the Lagrangian:

(33) $$L\left[\pi ,\;\gamma ,\;\lambda \right]=D_{\mathrm{KL}}\left[\pi \;∥\;\pi_{0}\right]+\gamma \left(\sum_{a}\pi \left(a\right)G\left(a\right)-C\right)+\lambda \left(\sum_{a}\pi \left(a\right)-1\right)$$

Differentiating L with respect to π(a) and setting the derivative to zero gives the stationarity condition:

$$\begin{array}{cc} \frac{\partial L}{\partial \pi (a)} & =\left(\ln\frac{\pi (a)}{\pi_{0}\left(a\right)}+1\right)+\gamma G\left(a\right)+\lambda =0\\ \Rightarrow \;\pi (a) & \propto \pi_{0}\left(a\right)\exp\left[-\gamma G(a)\right] \end{array}$$

Normalizing yields the optimal posterior policy defined in Equation (16). If $\pi_{0}$ is uniform, this reduces to a maximum entropy policy.

The two optimization problems in Equations (30) and (32) are formally dual. In the resource-rational formulation, expected cost is minimized subject to a fixed bound on information divergence. In the minimum-divergence formulation, divergence is minimized subject to a fixed bound on expected cost. These are mirror problems: each constrains the quantity that the other minimizes. Although their Lagrangians differ in form, both reduce to the same unconstrained objective once expressed through the precision parameter γ. Under convexity and Slater’s condition, strong duality holds, so the constrained problems, their Lagrangian duals, and the penalized (unconstrained) formulation all yield the same optimal policy [120].

The precision parameter γ encodes the marginal value of the active constraint. As the Lagrange multiplier in both formulations, it quantifies how the optimal objective value changes under infinitesimal relaxation of the constraint. Interpreted as a shadow price, γ captures the trade-off between performance and complexity. In the resource-rational (KL-constrained) formulation, γ is the marginal cost of information: the additional expected cost the agent accepts to reduce policy complexity by one nat. In the cost-constrained formulation, its reciprocal, 1/γ, represents the marginal cost of performance: the additional divergence the agent tolerates to lower expected cost by one unit. In both cases, the optimal policy satisfies a marginal equilibrium where the cost of relaxing a constraint equals the performance gain that deviation provides.

### 5.2. Robust Bayes Decisions and MaxEnt

Grünwald and Dawid [57] establish a key connection in MaxEnt decision theory by casting entropy as a special case of cross-entropy. Given distributions p∈P and q∈Q, the cross-entropy is defined as

(34) $$H\left[p,\;q\right]=-\sum_{s}p\left(s\right)\ln q\left(s\right)$$

Entropy H[p] emerges as the value of the optimization problem:

(35) $$H\left[p\right]=\underset{p\in P}{\sup}\underset{q\in Q}{\inf}H\left[p,\;q\right]$$

This identity follows from the information inequality [111], which guarantees that the infimum is achieved at q(s)=p(s).

Grünwald and Dawid show that this entropy formulation arises naturally from a minimax game. A decision-maker selects q∈Q to minimize expected log-loss, while nature selects p∈P to maximize it, subject to constraints. This setup yields the fundamental minimax identity:

(36) $$\underset{p\in P}{\sup}\underset{q\in Q}{\inf}H\left[p,\;q\right]=\underset{q\in Q}{\inf}\underset{p\in P}{\sup}H\left[p,\;q\right]$$

This result underlines a central duality linking Bayesian decision theory and the maximum entropy principle. The left-hand side expresses MaxEnt, choosing the distribution that maximizes entropy under constraints. The right-hand side expresses robust Bayes, choosing the predictive strategy that minimizes worst-case expected loss. This equivalence grounds maximum entropy methods in decision theory and shows that MaxEnt distributions solve robust optimization problems.

### 5.3. Applications and Extensions

Recent work has extended MaxEnt to several promising applications. One is MaxEnt inverse reinforcement learning, which addresses settings where the goal is to infer an agent’s reward function from observed behavior [121]. MaxEnt also plays a central role in an alternative derivation of the Quantal Response Equilibrium (QRE) [122,123,124,125] in game theory. In this alternative, two agents with classical expected utility functions maximize their entropies subject to expected utility constraints [126,127].

#### 5.3.1. Catastrophe Theory Meets Game Theory via MaxEnt

Harris et al. [128] proved that the bifurcations arising in QRE, first formalized via maximum entropy by Wolpert and Harré [126,127], are formally elementary catastrophes in the sense of Thom [129]. This was the first rigorous demonstration that boundedly rational decision theory implicitly defines the smooth potential functions required to classify bifurcations using Thom’s framework. Their result provides an alternative to potential game approaches [130,131,132].

Leonardos et al. [131] extended this MaxEnt perspective to reinforcement learning in multi-agent settings, uncovering further connections between learning dynamics and catastrophic transitions. These developments respond to earlier calls by Rosser Jr. [133] to reintroduce catastrophe theory into mainstream economics. They also align with recent information-theoretic analyses of critical transitions in economic systems, including market crashes [134], housing market dynamics [135], and interdisciplinary models of decision-making [94].

#### 5.3.2. Maximum Entropy Models for Neural Control and Inference

Savin et al. [136] formulate neural control as a constrained optimization problem, in which MaxEnt principles guide the selection of stimulation protocols under uncertainty. The controller selects inputs that satisfy empirical constraints while maximizing entropy, yielding control strategies that are robust to unmeasured or unknown parameters. This formulation treats neural control as a special case of Jaynes’ generalized inference problem: the goal is to identify the least-committal distribution over control signals consistent with observed neural dynamics.

This perspective emphasizes a broader insight: MaxEnt methods serve both inferential and normative roles in neuroscience. For example, they have been used to infer functional connectivity from limited neural recordings, often capturing higher-order correlations in population activity [137,138]. In this way, MaxEnt links microscopic variability to macroscopic structure, supporting robust inference and principled control within a unified framework.

## 6. Rate-Distortion Theory

Rate-distortion theory formalizes the trade-off between compression and reconstruction accuracy in communication systems [139,140,141]. Given a source random variable o∼p(o), the goal is to encode it into a compressed representation, or *code*, *s*, from which the original signal can be approximately reconstructed as $\hat{o}$. This process is governed by two conditional distributions: an *encoder* p(s∣o) that maps the source to the code, and a *decoder* $p(\hat{o}|s)$ that maps the code to a reconstruction [111]. Reconstruction quality is measured by a non-negative *distortion function* $d(o,\hat{o})$. The degree of compression is quantified by the *information rate* I[o;s], the mutual information between source and code. Compression is lossless when I[o;s]=H[o], meaning the original symbol can be recovered with certainty. When I[o;s]<H[o], the code discards source information and compression is lossy.

In this section, we show that action selection in active inference can be expressed as a rate–distortion problem: the expected free energy acts as the distortion term, while the KL divergence from prior habits sets an upper bound on the information rate. Under habit learning, this bound becomes tight.

### 6.1. Classical Rate-Distortion

The rate-distortion objective seeks an encoder–decoder pair that minimizes the information rate, subject to a constraint on expected distortion:

(37) $$\begin{array}{cc} \min_{p\left(s|o\right),p\left(\hat{o}|s\right)}\; & I[o;s]\\ \mathrm{subject}\;\mathrm{to}\; & E_{p(o,s,\hat{o})}\left[d\left(o,\;\hat{o}\right)\right]\leq D \end{array}$$

where the expectation is taken over the joint distribution $p\left(o,\;s,\;\hat{o}\right)=p\left(o\right)p\left(s|o\right)p\left(\hat{o}|s\right)$.

This constrained problem can be rewritten by introducing a Lagrangian:

(38) $$L[p]=I\left[o;s\right]+\beta \;E_{p(o,s,\hat{o})}\left[d\left(o,\;\hat{o}\right)\right]$$

where β≥0 controls the trade-off between rate and distortion.

Minimizing this Lagrangian over encoder and decoder distributions yields a parametric family of solutions indexed by β, each corresponding to a point on the rate-distortion curve:

(39) $$R\left(D\right)=\min_{p\left(s|o\right),\;p\left(\hat{o}|s\right):\;E\left[d\left(o,\hat{o}\right)\right]\leq D}I\left[o;s\right]$$

This function characterizes the boundary of the *rate-distortion region*: the set of all achievable pairs (R, D). The curve is convex and non-increasing in *D*, with each point representing the minimal information rate required to achieve expected distortion no greater than *D*.

### 6.2. Action Selection as a Rate-Distortion Trade-Off

Action selection admits a formal analogy to classical rate-distortion theory (Table 2). The central idea is that an agent must balance two competing objectives: minimizing the information rate (complexity) of its policy while maintaining acceptable distortion (cost). Policy complexity is quantified by the mutual information between states and actions, while performance is penalized through a cost function that defines distortion. This trade-off is governed by a Lagrangian objective, where a precision parameter regulates the balance between policy complexity and performance. As the parameter varies, it traces a Pareto frontier of optimal trade-offs, analogous to the rate-distortion curve in Equation (39).

This formulation has two close variants, developed in parallel, differing in how they treat the information rate.

#### 6.2.1. Self-Consistent Formulation

In the first variant, policy complexity is defined as the mutual information I[s; a] between states and actions [95,142,143]. A more complex policy selects actions that are more precisely tailored to each specific state. The objective is to minimize this complexity while keeping the expected cost below a threshold.

(40) $$\begin{array}{cc} \min_{\pi (a|s)}\; & I[s;\;a]\\ \mathrm{subject}\;\mathrm{to}\; & E_{p(s)\pi (a|s)}\left[G\left(s,\;a\right)\right]\leq C \end{array}$$

Solving this problem yields a fixed-point equation involving the marginal action distribution $\pi \left(a\right)=\sum_{s}p\left(s\right)\;\pi \left(a|s\right)$, which appears on both sides and must be computed self-consistently. This structure mirrors the Blahut–Arimoto algorithm [144,145] from classical rate-distortion theory, where π(a∣s) functions as an encoder and π(a) as the optimized marginal distribution over codewords:

(41) $$\pi^{*}\left(a|s\right)\propto \pi \left(a\right)\;\exp\left(-\gamma \;G(s,\;a)\right)$$

#### 6.2.2. Variational Formulation

The second variant is associated with active inference. This formulation simplifies the complexity term by replacing mutual information I[s; a] with a variational upper bound: the expected KL divergence between the agent’s policy and a fixed prior policy $\pi_{0}\left(a\right)$.

(42) $$\begin{array}{cc} \min_{\pi (a|s)}\; & E_{p(s)}\left[D_{\mathrm{KL}}\left[\pi \left(a|s\right)\;∥\;\pi_{0}\left(a\right)\right]\right]\\ \mathrm{subject}\;\mathrm{to}\; & E_{p(s)\pi (a|s)}\left[G\left(s,\;a\right)\right]\leq C \end{array}$$

This problem has a closed-form solution:

(43) $$\pi^{*}\left(a|s\right)\propto \pi_{0}\left(a\right)\;\exp\left(-\gamma \;G(s,\;a)\right)$$

Here, the agent selects actions by inferring a posterior policy π(a∣s) that minimizes expected free energy relative to a prior $\pi_{0}\left(a\right)$ given by its generative model.

#### 6.2.3. Equivalence via Habit Learning

The variational objective in (42) upper-bounds the true mutual information in (40) [146]. Their gap, the marginal-to-prior slack, arises from misalignment between the policy prior and the induced action marginal.

(44) $$E_{p(s)}\;\left[D_{\mathrm{KL}}\;\left[\pi \left(a|s\right)\;∥\;\pi_{0}\left(a\right)\right]\right]=I\left[s;\;a\right]+\underset{\mathrm{Marginal}–\mathrm{to}–\mathrm{prior}\;\mathrm{slack}}{\underset{︸}{D_{\mathrm{KL}}\;\left[\pi \left(a\right)\;∥\;\pi_{0}\left(a\right)\right]}}$$

As long as this divergence is nonzero, the resulting policy is only an approximation to the self-consistent optimum.

Minimizing the variational objective with respect to the prior removes the slack:

(45) $$\pi_{0}\left(a\right)=\pi \left(a\right)=\sum_{s}p\left(s\right)\;\pi \left(a|s\right)$$

which collapses the variational bound to the exact mutual-information formulation and restores the Blahut–Arimoto fixed point:

(46) $$\pi^{*}\left(a|s\right)\propto \pi \left(a\right)\;\exp\;\left[-\gamma \;G(s,a)\right]$$

Active inference does not perform this minimization analytically. Instead, it treats the prior $\pi_{0}\left(a\right)$ as a *habit distribution* shaped by past behavior. The agent recursively updates this prior over time, gradually reducing the marginal-to-prior divergence. This process, known as *policy caching* [38], entails selecting actions under the current habit-based prior, observing the resulting marginal distribution over actions, and updating the Dirichlet prior by accumulating expected action counts. Over repeated interactions, the prior increasingly reflects the agent’s own behavioral statistics. Under stationarity assumptions, this iterative refinement aligns the prior with the marginal, driving the system toward the fixed point where the marginal-to-prior divergence vanishes [38,146,147].

Finally, a related line of work applies rate–distortion ideas to perception and representation learning rather than policy selection [95,142,148]. These approaches typically appeal to an *information bottleneck* principle [149,150,151]: introduce a relevance variable *r* and seek a representation *s* that maximizes I[s;r] subject to an upper bound on I[s;o], so that *s* retains only task-relevant information about observations *o*. Different choices of *r* recover different perceptual objectives: future observations [142], task-specific queries such as class labels [148], or future reward [95]. In the latter two cases, the learned representations are explicitly action-centric, organized around their downstream role in guiding behavior. While this literature shares the same variational structure as the policy-centric formulation, it lies outside the scope of the present review.

## 7. Stochastic Optimal Control

Stochastic optimal control studies how agents make sequential decisions under uncertainty by minimizing cumulative cost. Classical approaches solve this problem with dynamic programming, using Bellman’s principle of optimality [152,153]. A more recent line of work reframes certain control problems as probabilistic inference. This control as inference view encodes costs as exponential likelihoods on trajectories and recovers policies as posterior distributions conditioned on desired outcomes [154]. Control as inference is itself a special case of active inference [155], which replaces cost with expected free energy and represents preferences directly as priors over observations. This section develops these three perspectives in turn: classical dynamic programming, control as inference, and their extension to active inference.

### 7.1. Classical Stochastic Optimal Control

Classical stochastic optimal control solves a fully observed Markov decision process (MDP) by computing a value function that satisfies the Bellman optimality equation and extracting the optimal stationary policy [153,156].

An MDP is defined by state space S, action space A, transition kernel $T(s^{\prime }|s,\;a)$, stage cost c(s, a), and discount factor δ∈[0, 1) [157]. The central object is the *optimal state-value function* $J^{*}\left(s\right)$, which gives the minimal expected cumulative discounted cost when starting from state *s* and following the optimal policy:

(47) $$J^{*}\left(s\right):=\min_{\pi }\;E_{\pi }\;\left[\sum_{t=0}^{\infty }\delta^{t}c\left(s_{t},\;a_{t}\right)\;|\;s_{0}=s\right]$$

The key relation that characterizes the optimal value function is the *Bellman optimality equation* [158]:

(48) $$J^{*}\left(s\right)=\min_{a\in A}\left[c\left(s,\;a\right)+\delta \sum_{s^{\prime }\in S}T\left(s^{\prime }|s,\;a\right)\;J^{*}\left(s^{\prime }\right)\right]$$

In each state *s*, the agent chooses the action that minimizes immediate cost plus the discounted cost-to-go. Because this principle applies to every state, the value function must satisfy a global system of self-consistent equations. Repeated application of the Bellman update enforces this consistency and drives any initial guess toward the unique fixed point, the optimal value function $J^{*}$ [159].

The *optimal action-value function* $Q^{*}\left(s,\;a\right)$ is the cost of taking *a* in *s* and then following the optimal policy [160]:

(49) $$Q^{*}\left(s,\;a\right)=c\left(s,\;a\right)+\delta \sum_{s^{\prime }\in S}T\left(s^{\prime }|s,\;a\right)\;J^{*}\left(s^{\prime }\right)$$

By construction, $J^{*}\left(s\right)$ equals the minimum over actions of $Q^{*}\left(s,\;a\right)$:

(50) $$J^{*}\left(s\right)=\min_{a\in A}Q^{*}\left(s,\;a\right)$$

Substituting this relation into (49) yields the recursive Bellman equation for $Q^{*}$:

(51) $$Q^{*}\left(s,\;a\right)=c\left(s,\;a\right)+\delta \sum_{s^{\prime }\in S}T\left(s^{\prime }|s,\;a\right)\;\min_{a^{\prime }\in A}Q^{*}\left(s^{\prime },\;a^{\prime }\right)$$

Intuitively, an action earns its value from its immediate cost plus the discounted value of the best actions available in the next state [157].

The *optimal stationary policy* follows directly from minimization:

(52) $$\pi^{*}\left(s\right)\in \arg\min_{a\in A}Q^{*}\left(s,\;a\right)$$

Once $Q^{*}$ is known, the agent acts optimally by selecting in each state the action whose $Q^{*}\left(s,\;a\right)$ value is minimal.

### 7.2. Control as Inference

Control as inference reframes stochastic optimal control as probabilistic inference. Instead of minimizing cost directly, it defines a generative model in which low-cost trajectories are exponentially more probable. Optimal policies are then recovered by inference in this model [154,161,162,163,164,165].

The prior trajectory distribution under a policy prior is

(53) $$p\left(\tau \right)=p\left(s_{0}\right)\prod_{t=0}^{\infty }p\left(a_{t}|s_{t}\right)\;T\left(s_{t+1}|s_{t},\;a_{t}\right)$$

where $p(s_{0})$ is the initial state distribution, p(a∣s) is the prior policy, and T is the transition kernel.

Optimality is expressed by tilting this prior with an exponential weight over cumulative cost:

(54) $$p\left(\tau |O=1\right)\;\propto \;p\left(\tau \right)\;\exp\;\left[-\gamma \sum_{t=0}^{\infty }\delta^{t}c\left(s_{t},\;a_{t}\right)\right]$$

where O is a binary optimality variable indicating whether a trajectory is optimal. Conditioning on O=1 reweights the prior trajectory distribution toward lower-cost trajectories. Trajectories with lower cumulative cost gain higher probability, paralleling statistical physics where paths are weighted by exp(−action). Here the cumulative cost plays the role of physical action [40].

To normalize this distribution, one must compute the sum of all the weights, which defines the *desirability function*, also known as the partition function:

(55) $$Z\left(s\right)=\sum_{\tau \;|\;s_{0}=s}p\left(\tau \right)\;\exp\;\left[-\gamma \sum_{t=0}^{\infty }\delta^{t}c\left(s_{t},\;a_{t}\right)\right]$$

which sums over trajectories starting at *s*. In continuous settings, this becomes a path integral, so solving the control problem amounts to computing a partition function over weighted trajectories.

Direct computation of Z(s) is intractable, but its logarithm defines a useful object. The *soft state-value function* [166] is

(56) $$J\left(s\right)=-\frac{1}{\gamma }\ln Z\left(s\right)$$

This is the negative log-partition function, analogous to free energy in statistical mechanics (and distinct from the variational free energy used in active inference).

This definition leads to a recursion that generalizes the Bellman equations [162]. The classical case used a min operator,

(57) $$J^{*}\left(s\right)=\min_{a\in A}\left[c\left(s,a\right)+\delta \sum_{s^{\prime }}T\left(s^{\prime }|s,\;a\right)\;J^{*}\left(s^{\prime }\right)\right]$$

where only the best action contributes. The soft analogue replaces this with a log-sum-exp [166]

(58) $$J\left(s\right)=-\frac{1}{\gamma }\ln\sum_{a\in A}p\left(a|s\right)\;\exp\;\left(-\gamma [c\left(s,\;a\right)+\delta \sum_{s^{\prime }}T\left(s^{\prime }|s,\;a\right)\;J\left(s^{\prime }\right)]\right)$$

Defining the *soft action-value function*

(59) $$Q\left(s,\;a\right)=c\left(s,\;a\right)+\delta \sum_{s^{\prime }}T\left(s^{\prime }|s,\;a\right)\;J\left(s^{\prime }\right)$$

shows that J(s) is the log-sum-exp of Q(s, a) values weighted by the action prior p(a∣s). As γ→∞, the exponential sharply penalizes suboptimal terms and the equations reduce to the classical Bellman form.

The corresponding optimal policy is a Boltzmann distribution over soft action-values:

(60) $$\pi^{*}\left(a|s\right)=\frac{p(a|s)\;\exp[-\gamma Q(s,\;a)]}{\sum_{a^{\prime }}p\left(a^{\prime }|s\right)\;\exp\left[-\gamma Q\left(s,a^{\prime }\right)\right]}$$

This recovers the greedy deterministic policy of classical control when γ→∞ and p(·∣s) is uniform. For finite γ, it yields a stochastic policy that distributes probability across actions in proportion to their soft values.

### 7.3. Control of Observations

Active inference can be aligned with control as inference by treating control as the regulation of observations [155]. In this view, both past sensory data and desired future outcomes are incorporated into a single, time-indexed generative model. Inference thus recovers both state estimation for t<k and action selection for t≥k within one probabilistic framework.

The joint generative model over trajectories is

(61) $$p\left(\tau \right)\;=\;p\left(s_{0}\right)\;\prod_{t=0}^{\infty }p\left(a_{t}|s_{t}\right)\;p\left(o_{t}|s_{t},\;a_{t}\right)\;T\left(s_{t+1}|s_{t},\;a_{t}\right)$$

Here $p(s_{0})$ is the initial state distribution, p(a∣s) the prior policy, $p(o_{t}|s_{t},\;a_{t})$ the observation likelihood, and T the transition kernel.

The horizon is partitioned at *k*. For t<k, the agent employs the standard observation model

(62) $$p\left(o_{t}|s_{t},\;a_{t}\right)=p_{\mathrm{obs}}\left(o_{t}|s_{t},\;a_{t}\right)$$

so inference reduces to variational state estimation.

For t≥k, preferences over future outcomes are expressed through a likelihood derived from a preference distribution,

(63) $$p^{*}\left(o_{t}\right)\;\propto \;\exp\left[-\gamma \;c\left(o_{t}\right)\right]$$

where $c(o_{t})$ is a cost function and γ a precision parameter. Low-cost outcomes receive high prior probability, making explicit the equivalence between preferences and costs. Embedding this into the generative model yields the soft likelihood

(64) $$p\left(o_{t}|s_{t},\;a_{t}\right)=\frac{1}{Z_{t}}\;\exp\left[-\gamma \;c\left(s_{t},a_{t}\right)\right]$$

where $Z_{t}$ is the partition function ensuring normalization. This recovers the optimality likelihood used in control as inference (cf. Equation (54)), but now applied directly to observations.

The inference problem is to approximate the posterior distribution

(65) $$p(s_{0:\infty },\;a_{0:\infty }|o_{0:k-1},\;o_{k:\infty }^{*})$$

which conditions on past observations and on future preferred outcomes. Because this posterior is generally intractable, we introduce a variational distribution $q(s_{0:\infty },\;a_{0:\infty })$ and minimize the divergence

(66) $$D_{\mathrm{KL}}\;\left[q\left(s_{0:\infty },\;a_{0:\infty }\right)\;∥\;p\left(s_{0:\infty },\;a_{0:\infty }|o_{0:k-1},\;o_{k:\infty }^{*}\right)\right]$$

This is equivalent to maximizing a lower bound on the log-evidence of outcomes, or equivalently minimizing the *variational free energy*.

Minimizing the variational free energy yields

(67) $$\min_{q}\;\sum_{t=0}^{k-1}F_{t}\left[q_{t},\;o_{t}\right]+\sum_{t=k}^{\infty }\delta^{t}F_{t}\left[q_{t},\;o_{t}^{*}\right]$$

with per-time-step contributions

(68) $$F_{t}\left[q_{t},\;{\tilde{o}}_{t}\right]=D_{\mathrm{KL}}\;\left[q_{t}\left(s_{t},\;a_{t}\right)\;∥\;p_{t}\left(s_{t}\right)\;p\left(a_{t}|s_{t}\right)\right]-E_{q_{t}}\;\left[\ln p({\tilde{o}}_{t}|s_{t},\;a_{t})\right]$$

Here $p_{t}\left(s_{t}\right)$ is the marginal state prior induced by $p(s_{0})$, p(a∣s), and T, and δ∈[0, 1) is a discount factor ensuring convergence of the infinite sum.

For t<k, ${\tilde{o}}_{t}=o_{t}$ corresponds to actual observations, so this objective reduces to standard state estimation. For t≥k, ${\tilde{o}}_{t}=o_{t}^{*}$ encodes preferences as pseudo-observations, so the objective recovers a KL-regularized control functional balancing fidelity to the policy prior with preference satisfaction.

In the linear–Gaussian case, with linear dynamics and Gaussian observation and preference models, this construction recovers Kalman filtering [167] for t<k and linear–quadratic regulation (LQR) [168] for t≥k. The control-of-observations framework therefore unifies estimation and control in a single probabilistic scheme, includes classical LQG as a special case, and extends naturally to nonlinear generative models.

## 8. Reinforcement Learning

Reinforcement learning is a framework for sequential decision-making in which agents learn to maximize expected cumulative reward by interacting with an environment [169]. This section connects RL with active inference by comparing maximum entropy reinforcement learning, deep actor–critic methods, and model-based approaches based on learned world models. We show that under a uniform reference policy, active inference recovers the soft-optimal policy of maximum entropy RL. More broadly, active inference replaces action-value functions with negative expected free energy and reframes policy optimization as a problem of Bayesian inference. We then compare model-based RL and active inference in how they represent uncertainty, plan over future states, and update their beliefs.

### 8.1. Classical Reinforcement Learning

Classical reinforcement learning solves a fully observed Markov decision process in a way directly analogous to stochastic optimal control, but with *rewards* instead of *costs*. While in Section 7.1 the objective was to minimize expected cumulative cost, here the agent seeks to maximize expected cumulative reward.

An MDP is defined by state space S, action space A, transition kernel $T(s^{\prime }|s,\;a)$, reward function r(s, a), and discount factor δ∈[0, 1). The central object is the *optimal state-value function* $V^{*}\left(s\right)$, which gives the maximal expected cumulative discounted reward when starting from state *s* and following the optimal policy [170]:

(69) $$V^{*}\left(s\right):=\max_{\pi }\;E_{\pi }\;\left[\sum_{t=0}^{\infty }\delta^{t}r\left(s_{t},\;a_{t}\right)\;|\;s_{0}=s\right]$$

This mirrors the role of $J^{*}\left(s\right)$ in stochastic control, but replaces minimization of costs with maximization of rewards.

The key relation that characterizes the optimal value function is the *Bellman optimality equation* [158]:

(70) $$V^{*}\left(s\right)=\max_{a\in A}\left[r\left(s,a\right)+\delta \sum_{s^{\prime }\in S}T\left(s^{\prime }|s,a\right)\;V^{*}\left(s^{\prime }\right)\right]$$

This is structurally identical to (48), except with “max” in place of “min.” The principle is the same: optimal value arises from immediate payoff plus the discounted continuation value of future states.

The *optimal action-value function* $Q^{*}\left(s,\;a\right)$ quantifies the reward of committing to a particular action *a* now and then acting optimally afterward:

(71) $$Q^{*}\left(s,\;a\right)=r\left(s,\;a\right)+\delta \sum_{s^{\prime }\in S}T\left(s^{\prime }|s,\;a\right)\;V^{*}\left(s^{\prime }\right)$$

This is the reward-maximizing counterpart to the cost-based definition (cf. (49)).

By construction, $V^{*}\left(s\right)$ equals the maximum over actions of $Q^{*}\left(s,\;a\right)$:

(72) $$V^{*}\left(s\right)=\max_{a\in A}Q^{*}\left(s,\;a\right)$$

Substituting this relation into (71) yields the recursive Bellman equation for $Q^{*}$:

(73) $$Q^{*}\left(s,\;a\right)=r\left(s,\;a\right)+\delta \sum_{s^{\prime }\in S}T\left(s^{\prime }|s,\;a\right)\;\max_{a^{\prime }\in A}Q^{*}\left(s^{\prime },\;a^{\prime }\right)$$

As before, the recursion expresses the idea that an action’s value combines immediate reward with the value of the best continuation.

The *optimal stationary policy* follows directly from maximization:

(74) $$\pi^{*}\left(s\right)\in \arg\max_{a\in A}Q^{*}\left(s,\;a\right)$$

In parallel with (52), the policy is recovered by a simple greedy choice, but now the agent selects the action with maximal long-run return rather than minimal long-run cost.

### 8.2. Maximum Entropy Reinforcement Learning

Maximum entropy reinforcement learning extends the classical value-based formulation by adding an entropy bonus to the return [121,171]. The agent is no longer concerned only with maximizing expected cumulative reward, but also with maintaining stochasticity in its policy. This turns the purely exploitative goal of classical RL into a balance between reward and entropy maximization.

Formally, the agent maximizes the *entropy-regularized return*:

(75) $$R_{t}=\sum_{k=0}^{\infty }\delta^{k}\left[r\left(s_{t+k},\;a_{t+k}\right)+\frac{1}{\gamma }\;H\left[\pi \left(\cdot |s_{t+k}\right)\right]\right]$$

where γ>0 plays the role of an inverse temperature. Large γ sharpens the policy toward determinism, while small γ increases the weight of the entropy term.

Unlike in classical RL (Section 8.1), the value of a state now depends directly on the distribution of the policy. This forces us to introduce explicitly *policy-dependent soft value functions* [121].

The *soft action-value function* evaluates the reward of taking an action *a* in state *s*, then following policy π:

(76) $$Q^{\pi }\left(s,\;a\right)=r\left(s,\;a\right)+\delta \;E_{s^{\prime }∼T\left(\cdot |s,\;a\right)}\left[V^{\pi }\left(s^{\prime }\right)\right]$$

The *soft state-value function* evaluates the expected return of starting in *s* and sampling actions from π:

(77) $$V^{\pi }\left(s\right)=E_{a∼\pi }\left[Q^{\pi }\left(s,\;a\right)-\frac{1}{\gamma }\ln\pi \left(a|s\right)\right]$$

Here the second term, $-\frac{1}{\gamma }\ln\pi \left(a|s\right)$, shows explicitly how the policy’s entropy contributes to the state value. This is the crucial difference: in classical RL, $V^{\pi }$ depended on π only because it mapped states to actions; in maximum entropy RL, $V^{\pi }$ depends directly on the distribution of π itself.

Optimizing over all policies yields the *soft optimal value functions*. The soft optimal state-value function replaces the hard maximum of the Bellman optimality equation with a log-sum-exp operator [162]:

(78) $$V^{*}\left(s\right)=\frac{1}{\gamma }\;\ln\sum_{a\in A}\exp\;\left(\gamma \;Q^{*}\left(s,\;a\right)\right)$$

which corresponds to the *exponential certainty equivalent* of the *Q*-values under exponential utility, with γ controlling risk sensitivity [172,173]. In the limit γ→∞, the entropy term vanishes and this reduces to the classical Bellman Equation (70).

Finally, the optimal policy is recovered in closed form as a Boltzmann distribution over $Q^{*}$:

(79) $$\pi^{*}\left(a|s\right)=\frac{\exp\;\left(\gamma \;Q^{*}\left(s,\;a\right)\right)}{\sum_{a^{\prime }}\exp\;\left(\gamma \;Q^{*}\left(s,\;a^{\prime }\right)\right)}$$

Viewed as an implicit Gibbs distribution over actions, this makes it clear that γ acts as a precision parameter, scaling the log-probability differences between actions. This stands in contrast to the greedy deterministic policy of classical RL (cf. (74)). Here, stochasticity is built into the solution: even the optimal policy preserves entropy, ensuring continual exploration [174,175]. Related precision-like gain parameters have been associated, in neurocomputational accounts of dopamine, with the scaling of temporal-difference reward-prediction errors in cortico-striatal circuits, providing a further link to biological reinforcement learning [176,177,178].

### 8.3. KL Regularization View

The entropy term in (75) can be reinterpreted as a KL divergence between the policy π(·∣s) and a prior distribution p(·∣s) [115,179]. In this setting, the KL term acts as a *regularizer*, a component added to the optimization objective to prevent overfitting or instability by constraining how far the learned policy may deviate from a reference distribution [180].

Maximizing reward plus entropy is equivalent, up to an additive constant, to maximizing reward minus a KL penalty:

(80) $$E_{a∼\pi }\left[r\left(s,\;a\right)\right]+\frac{1}{\gamma }H\left[\pi \left(\cdot |s\right)\right]≐E_{a∼\pi }\left[r\left(s,\;a\right)\right]-\frac{1}{\gamma }\;D_{\mathrm{KL}}\;\left[\pi (\cdot |s)\;∥\;p(\cdot |s)\right]$$

with p(·∣s) taken as the uniform distribution. In this case, encouraging high entropy is the same as penalizing deviation from uniformity.

More generally, one may choose a non-uniform prior p(·∣s) to bias exploration toward certain actions. This yields a *generalized maximum entropy objective*:

(81) $$R_{t}=\sum_{k=0}^{\infty }\delta^{k}\left[r\left(s_{t+k},\;a_{t+k}\right)-\frac{1}{\gamma }\;D_{\mathrm{KL}}\;\left[\pi \left(\cdot |s_{t+k}\right)\;∥\;p\left(\cdot |s_{t+k}\right)\right]\right]$$

This view shows maximum entropy RL as a special case of regularized RL with a uniform prior.

The corresponding optimal policy generalizes the Boltzmann distribution in (79). Instead of

(82) $$\pi^{*}\left(a|s\right)=\frac{\exp\;\left(\gamma \;Q^{*}\left(s,\;a\right)\right)}{\sum_{a^{\prime }}\exp\;\left(\gamma \;Q^{*}\left(s,\;a^{\prime }\right)\right)}$$

one recovers the *prior-weighted Boltzmann policy*:

(83) $$\pi^{*}\left(a|s\right)=\frac{p\left(a|s\right)\;\exp\;\left(\gamma \;Q^{*}\left(s,\;a\right)\right)}{\sum_{a^{\prime }}p\left(a^{\prime }|s\right)\;\exp\;\left(\gamma \;Q^{*}\left(s,\;a^{\prime }\right)\right)}$$

Here the prior p(a∣s) tilts the distribution toward preferred actions. If p(·∣s) is uniform, this reduces back to the maximum entropy solution (79). With a structured prior, the agent trades off maximizing $Q^{*}$ with staying close to *p*, effectively combining learned value with inductive biases encoded in the prior.

This prior-weighted Boltzmann form (83) is directly analogous to the optimal posterior policy in active inference (cf. (16)), where negative expected free energy G(a) replaces the action-value function and $\pi_{0}\left(a\right)$ plays the role of the prior policy. Seen this way, maximum entropy reinforcement learning with KL regularization can be interpreted as the control-theoretic counterpart of active inference, with both frameworks arriving at the same structural solution through different starting points.

### 8.4. Model-Free Learning

Maximum entropy reinforcement learning is often presented in a model-based formulation where the transition dynamics T are known and used to compute soft Bellman backups. In practice, many algorithms are model-free, learning value functions and policies directly from sampled trajectories without requiring access to the environment’s dynamics [181,182,183].

Within this framework, *Soft Q-learning* [181] learns a soft action-value function by bootstrapping from observed rewards and estimating the downstream value with a soft (log-sum-exp) maximum over actions. The resulting optimal policy is a Boltzmann distribution over the learned value function. *Soft Actor–Critic (SAC)* [182] generalizes this by learning both a stochastic policy and soft value estimates. The policy is optimized to maximize expected return while encouraging entropy, yielding a trade-off between performance and exploration controlled by a temperature parameter.

A conditional correspondence can be drawn to active inference when the negative expected free energy is identified with the learned soft Q-function. Under a uniform action prior, action selection then follows a Boltzmann distribution identical in form to the maximum entropy policy. In this view, model-free soft value learning implements an amortized approximation to inference over actions, recovering the same stochastic policies prescribed by active inference under a flat prior.

In multi-agent settings, KL-regularized policy search can maintain human-compatible behavior. The piKL algorithm [23] applies a no-regret update that improves expected reward through mirror-descent dynamics [184] and adds a behavioral cloning constraint that limits divergence from an anchor policy derived from human demonstrations. The KL penalty keeps the learned policy aligned with human conventions while allowing improvement through self-play and optimization.

### 8.5. Model-Based Learning

Model-based reinforcement learning (MBRL) incorporates an explicit model of the environment’s dynamics to support planning and decision-making. The theoretical limit of such an approach is AIXI [185], which defines Bayes-optimal behavior by combining Solomonoff induction [186] with sequential decision theory. Although incomputable and not a tractable algorithm for real-world problems, AIXI serves as a conceptual benchmark for fully Bayesian reinforcement learning. Practical MBRL methods approximate this ideal by maintaining and updating beliefs about environment dynamics from observed transitions [187], allowing agents to plan or to generate imagined trajectories for training policies and value functions. In principle, this Bayesian treatment of uncertainty supports principled exploration by quantifying and responding to uncertainty, though exact Bayes-adaptive planning is typically intractable at realistic scales [188].

Recent formulations reinterpret bounded planning as lossy Bayesian inference, where agents trade off expected utility against the information cost of representing or learning policies. This connects to the rate–distortion framework discussed in Section 6. For instance, Arumugam et al. [189] formalise this trade-off by treating learning targets as compressed representations that balance expected performance against representational cost, supporting capacity-sensitive exploration and approximate planning.

Contemporary deep model-based methods, such as Dreamer [190,191] and MBPO [192], learn a latent *world model* [193,194,195]: a compact representation of environment dynamics used to simulate imagined rollouts for policy and value learning. These models enable planning through internal simulation and bear a high-level structural resemblance to the generative models used in active inference, where agents select actions by minimizing expected free energy. However, the objectives and inference schemes differ.

While active inference adopts a variational objective (variational free energy and expected free energy), deep MBRL methods rely on amortized optimization: learned parametric mappings that approximate inference through gradient-based learning rather than explicit message passing [196]. Fixed-capacity architectures and approximate uncertainty propagation are design choices made for computational efficiency and scalability. While this forfeits exact Bayesian updates, these methods achieve strong empirical performance in high-dimensional settings [77,190,191,197,198]. The conceptual correspondence between active inference and frontier model-based RL therefore remains conditional: both use internal generative models, but they differ in objective function, treatment of uncertainty, and mechanisms of belief or policy updating.

## 9. Discussion

### 9.1. Formal Unification

Active inference can be situated within a broader class of variational formulations that share a common mathematical structure [28,114]. This general form optimizes an objective that combines expected loss with a regularization term penalizing deviation from a prior distribution:

(84) $$p^{*}=\arg\min_{p}\;E_{p}\left[L\right]+\frac{1}{\gamma }\;D\left(p∥p_{0}\right)$$

Here, L(x) denotes the loss or cost associated with outcome *x*, p(x) is the posterior or decision distribution, and $p_{0}\left(x\right)$ is the prior distribution encoding baseline beliefs or preferences. The inverse temperature parameter γ modulates the balance between minimizing expected loss and maintaining proximity to the prior.

This formulation yields an exponential-family solution derived from the convex duality between entropy and the log-sum-exp function, which defines the soft value or dual objective:

(85) $$p^{*}\left(x\right)=\frac{p_{0}\left(x\right)\;e^{-\gamma L(x)}}{Z},\;\;Z=\sum_{x}p_{0}\left(x\right)\;e^{-\gamma L(x)}$$

Several established frameworks instantiate this general structure:

- Resource-rational choice minimizes expected utility under an information cost, converging to Bayesian decision theory when information is costless.
- The maximum entropy principle maximizes entropy subject to an expected loss constraint.
- Rate-distortion theory minimizes distortion subject to an information constraint.
- Stochastic optimal control minimizes trajectory cost regularized by divergence from a prior policy.
- Soft reinforcement learning applies the same principle to state–action distributions.

Active inference corresponds to a particular instance in which the (pragmatic) loss function is given by a *negative log-likelihood* (NLL) or *log loss*, though this correspondence does not apply uniformly across all related frameworks (Table 3).

Active inference explicitly incorporates information gain as a component of its formulation, a feature not represented in the other frameworks. Thus, while active inference and these frameworks exhibit formal correspondences, they remain distinct in scope and emphasis.

### 9.2. Representational Constraints

The normative force of expected free energy minimization depends on the representational adequacy of the generative model. Two constraints are particularly important.

First, many Bayes-adaptive models assume stationary latent parameters [187]. In nonstationary environments, the posterior accumulates indefinitely, becoming overconfident and slow to adapt under drift [199]. Heuristic solutions impose finite memory via exponential discounting, sliding windows, or change point detection to cap effective information and restore responsiveness. Optimal solutions endogenize change through priors on latent dynamics so adaptation follows directly from the generative model.

Second, generative models that lack sufficient temporal depth will overestimate the entropy rate of the underlying generative process and underestimate its predictability [200,201]. In these cases, the belief state q(s) fails to capture all relevant information and the model treats unmodeled temporal dependencies as noise, leading to systematically suboptimal policies.

These limitations do not arise from the expected free energy objective itself but from representational choices that determine how well inference can track the true data-generating process.

### 9.3. Extensions

The expected free energy framework has been extended in several directions, including scale-free hierarchies [16,202], structure learning [203], meta-inference [204], and multi-agent formulations [205,206]. These developments offer coherent theoretical generalizations but remain subjects of ongoing algorithmic and empirical work. Hierarchical and scale-free models propose stacked generative structures that minimize expected free energy across nested temporal and spatial scales. They resemble hierarchical reinforcement learning architectures but differ in objectives and inference mechanisms [16]. Structure learning methods, including Bayesian model expansion and reduction, provide principled tools for adapting model complexity [203], though automated discovery at the scale achieved in modern deep reinforcement learning is still out of reach. Meta-inference, or *sophisticated inference*, broadens the normative scope by treating policy selection as inference over future belief trajectories, at the cost of higher computational and representational demands [204]. Multi-agent formulations extend the framework to systems involving other agents’ latent states or policies, connecting active inference to game-theoretic analysis [207,208]. Challenges of identifiability, equilibrium selection, and nonstationarity remain key targets for future investigation.

### 9.4. Computational Considerations

The recent literature on efficient implementations of active inference indicates that naive evaluation of expected free energy over policy trees, when combined with rich generative models, can quickly become computationally expensive and is typically only feasible for relatively small problems in practice [209,210]. Practical schemes seek to improve scalability by exploiting structure and approximation, for example through dynamic-programming style recursions for expected free energy [209], branching-time or tree-search formulations [210,211,212,213], contrastive latent representations [214], or message passing on factor graphs [215]. These approaches can yield substantial speedups and handle non-trivial control tasks in practice, but they do not alter the classical worst-case complexity of partially observed control problems: planning in finite-horizon POMDPs is PSPACE-complete [216], and, in general, certain infinite-horizon decision problems for POMDPs are undecidable [217].

### 9.5. Closing Remarks

Active inference’s expected free energy objective combines utility maximization, information gain, and resource constraints within a single variational framework. It treats inference, control, and decision as instances of the same convex optimization that balances accuracy and complexity. The various frameworks we explore differ in their priors, loss functions, and regularizers, so they are not formally identical, but exhibit strong structural correspondences. This perspective highlights a shared geometry linking probabilistic, control, and information-theoretic approaches. Active inference thus offers a coherent foundation for studying adaptive systems through variational principles, providing a common language for neuroscience, machine learning, and the cognitive and social sciences.

## Figures and Tables

**Table 1:** Comparison of action-selection objectives by included terms and policy form. *Symbols:* ↑ maximize; ↓ minimize; ✓ present; – absent.

Framework	Objective	Pragmatic Value	Entropy	Ambiguity	Stochastic Policy
Bayesian decision theory	↑ Expected utility	✓	–	–	–
Bounded rationality	↑ Expected utility	✓	–	–	✓
Maximum entropy	↑ Entropy	–	✓	–	✓
Rate-distortion	↓ Distortion	✓	✓	–	✓
Stochastic control	↓ Cost	✓	–	–	✓
Reinforcement learning	↑ Value	✓	–	–	✓
Active inference	↓ Expected free energy	✓	✓	✓	✓

**Table 2:** Rate-distortion analogies in action selection.

Element	Active Inference Interpretation	Rate-Distortion Role
s∼q(s)	State distribution	Source
π(a∣s)	Stochastic policy	Encoder
*a*	Action selected	Codeword
G(s, a)	Cost function	Distortion measure
I(s; a)	Policy complexity	Information rate
E[G(s, a)]	Expected performance loss	Expected distortion
γ	Precision parameter	Rate–distortion trade-off weight

**Table 3:** Presence of negative log-likelihood (NLL) loss and KL regularization across frameworks. *Symbols:* ✓ present in all standard formulations; – absent; ^†^ present as a special case. ^*^ In rate-distortion theory, the distortion measure becomes equivalent to an NLL only under a probabilistic interpretation.

Framework	NLL Loss	KL Regularizer
Bayesian decision theory	^†^	–
Resource-rational decision theory	^†^	✓
Maximum entropy	^†^	✓
Rate-distortion theory	^†^^*^	✓
Stochastic optimal control	^†^	^†^
Reinforcement learning	^†^	^†^
Active inference	✓	✓

## Data Availability

No new data were created or analyzed in this study. Data sharing is not applicable to this article.

## Abbreviations

The following abbreviations are used in this manuscript:

AIXI	Artificial intelligence with Solomonoff induction
EFE	Expected free energy
EVSI	Expected value of sample information
KL	Kullback-Leibler
LQG	Linear–quadratic–Gaussian
LQR	Linear–quadratic regulation
MaxEnt	Maximum entropy (principle)
MBPO	Model-based policy optimization
MBRL	Model-based reinforcement learning
MDP	Markov decision process
POMDP	Partially observable Markov decision process
QRE	Quantal response equilibrium
RL	Reinforcement learning
SAC	Soft actor–critic

## References

[1] Sajid N., Ball P.J., Parr T., Friston K.J. (2021). Active Inference: Demystified and Compared. Neural Comput..

[2] Parr T., Pezzulo G., Friston K.J. (2022). Active Inference: The Free Energy Principle in Mind, Brain, and Behavior.

[3] Friston K.J., Salvatori T., Isomura T., Tschantz A., Kiefer A., Verbelen T., Koudahl M., Paul A., Parr T., Razi A. (2025). Active Inference and Intentional Behavior. Neural Comput..

[4] Gottwald S., Braun D.A. (2020). The Two Kinds of Free Energy and the Bayesian Revolution. PLoS Comput. Biol..

[5] Blei D.M., Kucukelbir A., McAuliffe J.D. (2017). Variational Inference: A Review for Statisticians. J. Am. Stat. Assoc..

[6] Parr T., Friston K.J. (2019). Generalised Free Energy and Active Inference. Biol. Cybern..

[7] Costa L.D., Tenka S., Zhao D., Sajid N. (2024). Active Inference as a Model of Agency. arXiv.

[8] Friston K., Samothrakis S., Montague R. (2012). Active Inference and Agency: Optimal Control without Cost Functions. Biol. Cybern..

[9] Tschantz A., Millidge B., Seth A.K., Buckley C.L. (2020). Reinforcement Learning through Active Inference. arXiv.

[10] Ueltzhöffer K. (2018). Deep Active Inference. Biol. Cybern..

[11] Lanillos P., Meo C., Pezzato C., Meera A.A., Baioumy M., Ohata W., Tschantz A., Millidge B., Wisse M., Buckley C.L. (2021). Active Inference in Robotics and Artificial Agents: Survey and Challenges. arXiv.

[12] Friston K., FitzGerald T., Rigoli F., Schwartenbeck P., Pezzulo G. (2017). Active Inference: A Process Theory. Neural Comput..

[13] Da Costa L., Parr T., Sajid N., Veselic S., Neacsu V., Friston K. (2020). Active Inference on Discrete State-Spaces: A Synthesis. J. Math. Psychol..

[14] Smith R., Friston K.J., Whyte C.J. (2022). A Step-by-Step Tutorial on Active Inference and Its Application to Empirical Data. J. Math. Psychol..

[15] Pezzulo G., Parr T., Friston K. (2024). Active Inference as a Theory of Sentient Behavior. Biol. Psychol..

[16] Friston K., Heins C., Verbelen T., Da Costa L., Salvatori T., Markovic D., Tschantz A., Koudahl M., Buckley C., Parr T. (2025). From Pixels to Planning: Scale-Free Active Inference. Front. Netw. Physiol..

[17] Friston K. (2010). The Free-Energy Principle: A Unified Brain Theory?. Nat. Rev. Neurosci..

[18] Friston K. (2019). A Free Energy Principle for a Particular Physics. arXiv.

[19] Buckley C.L., Kim C.S., McGregor S., Seth A.K. (2017). The Free Energy Principle for Action and Perception: A Mathematical Review. J. Math. Psychol..

[20] Ramstead M.J.D., Sakthivadivel D.A.R., Heins C., Koudahl M., Millidge B., Da Costa L., Klein B., Friston K.J. (2023). On Bayesian Mechanics: A Physics of and by Beliefs. Interface Focus.

[21] Friston K., Da Costa L., Sakthivadivel D.A.R., Heins C., Pavliotis G.A., Ramstead M., Parr T. (2023). Path Integrals, Particular Kinds, and Strange Things. Phys. Life Rev..

[22] Friston K., Da Costa L., Sajid N., Heins C., Ueltzhöffer K., Pavliotis G.A., Parr T. (2023). The Free Energy Principle Made Simpler but Not Too Simple. Phys. Rep..

[23] Jacob A.P., Wu D.J., Farina G., Lerer A., Hu H., Bakhtin A., Andreas J., Brown N. Modeling Strong and Human-Like Gameplay with KL-Regularized Search. Proceedings of the 39th International Conference on Machine Learning, PMLR.

[24] Friston K., Heins C., Ueltzhöffer K., Da Costa L., Parr T. (2021). Stochastic Chaos and Markov Blankets. Entropy.

[25] Sakthivadivel D.A.R. (2022). Weak Markov Blankets in High-Dimensional, Sparsely-Coupled Random Dynamical Systems. arXiv.

[26] Wainwright M.J., Jordan M.I. (2008). Graphical Models, Exponential Families, and Variational Inference. Found. Trends^®^ Mach. Learn..

[27] Honkela A., Valpola H. (2004). Variational Learning and Bits-Back Coding: An Information-Theoretic View to Bayesian Learning. IEEE Trans. Neural Netw..

[28] Knoblauch J., Jewson J., Damoulas T. (2022). An Optimization-centric View on Bayes’ Rule: Reviewing and Generalizing Variational Inference. J. Mach. Learn. Res..

[29] Friston K.J., Lin M., Frith C.D., Pezzulo G., Hobson J.A., Ondobaka S. (2017). Active Inference, Curiosity and Insight. Neural Comput..

[30] Parr T., Friston K.J. (2017). Uncertainty, Epistemics and Active Inference. J. R. Soc. Interface.

[31] Kaelbling L.P., Littman M.L., Cassandra A.R. (1998). Planning and Acting in Partially Observable Stochastic Domains. Artif. Intell..

[32] Murphy K.P. (2002). Dynamic Bayesian Networks: Representation, Inference and Learning. Ph.D. Thesis.

[33] Åström K. (1965). Optimal Control of Markov Processes with Incomplete State Information. J. Math. Anal. Appl..

[34] van Oostrum J., Langer C., Ay N. (2025). A Concise Mathematical Description of Active Inference in Discrete Time. J. Math. Psychol..

[35] Robbins H. (1952). Some Aspects of the Sequential Design of Experiments. Bull. Am. Math. Soc..

[36] Russell S., Norvig P. (2020). Artificial Intelligence: A Modern Approach.

[37] Friston K., Rigoli F., Ognibene D., Mathys C., Fitzgerald T., Pezzulo G. (2015). Active Inference and Epistemic Value. Cogn. Neurosci..

[38] Friston K., FitzGerald T., Rigoli F., Schwartenbeck P., O’Doherty J., Pezzulo G. (2016). Active Inference and Learning. Neurosci. Biobehav. Rev..

[39] Limanowski J., Adams R.A., Kilner J., Parr T. (2024). The Many Roles of Precision in Action. Entropy.

[40] Kappen H.J. (2005). Path Integrals and Symmetry Breaking for Optimal Control Theory. J. Stat. Mech. Theory Exp..

[41] Savage L. (1954). The Foundations of Statistics.

[42] Berger J.O. (1985). Statistical Decision Theory and Bayesian Analysis.

[43] Chaloner K., Verdinelli I. (1995). Bayesian Experimental Design: A Review. Stat. Sci..

[44] Ryan E., Drovandi C., McGree J., Pettitt T. (2016). A Review of Modern Computational Algorithms for Bayesian Optimal Design. Int. Stat. Rev..

[45] Sajid N., Costa L.D., Parr T., Friston K., Wu C.M., Schulz E., Cogliati Dezza I. (2022). Active Inference, Bayesian Optimal Design, and Expected Utility. The Drive for Knowledge: The Science of Human Information Seeking.

[46] Da Costa L., Sajid N., Parr T., Friston K., Smith R. (2023). Reward Maximization Through Discrete Active Inference. Neural Comput..

[47] von Neumann J., Morgenstern O. (2007). Theory of Games and Economic Behavior: 60th Anniversary Commemorative Edition.

[48] Candeal J.C., De Miguel J.R., Indurain E., Mehta G.B. (2001). Utility and Entropy. Econ. Theory.

[49] Ortega P.A., Braun D.A. A Conversion between Utility and Information. Proceedings of the 3rd Conference on Artificial General Intelligence (AGI-10).

[50] Savage L.J. (1971). Elicitation of Personal Probabilities and Expectations. J. Am. Stat. Assoc..

[51] Gneiting T., Raftery A.E. (2007). Strictly Proper Scoring Rules, Prediction, and Estimation. J. Am. Stat. Assoc..

[52] Dawid A.P. (2007). The Geometry of Proper Scoring Rules. Ann. Inst. Stat. Math..

[53] Jose V.R.R., Nau R.F., Winkler R.L. (2008). Scoring Rules, Generalized Entropy, and Utility Maximization. Oper. Res..

[54] Amari S.i. (2016). Information Geometry and Its Applications.

[55] Chambers C.P., Healy P.J., Lambert N.S. (2019). Proper Scoring Rules with General Preferences: A Dual Characterization of Optimal Reports. Games Econ. Behav..

[56] Grünwald P.D. (2007). The Minimum Description Length Principle.

[57] Grünwald P.D., Dawid A.P. (2004). Game Theory, Maximum Entropy, Minimum Discrepancy and Robust Bayesian Decision Theory. Ann. Stat..

[58] Lindley D.V. (1956). On a Measure of the Information Provided by an Experiment. Ann. Math. Stat..

[59] Bernardo J.M. (1979). Expected Information as Expected Utility. Ann. Stat..

[60] MacKay D.J.C. (2019). Information Theory, Inference and Learning Algorithms.

[61] Raiffa H., Schlaifer R. (2000). Applied Statistical Decision Theory.

[62] Kidd C., Hayden B.Y. (2015). The Psychology and Neuroscience of Curiosity. Neuron.

[63] Sharot T., Sunstein C.R. (2020). How People Decide What They Want to Know. Nat. Hum. Behav..

[64] Gottlieb J., Oudeyer P.Y. (2018). Towards a Neuroscience of Active Sampling and Curiosity. Nat. Rev. Neurosci..

[65] Bach D.R., Dolan R.J. (2012). Knowing How Much You Don’t Know: A Neural Organization of Uncertainty Estimates. Nat. Rev. Neurosci..

[66] Oudeyer P.Y., Kaplan F. (2007). What Is Intrinsic Motivation? A Typology of Computational Approaches. Front. Neurorobot..

[67] Chentanez N., Barto A., Singh S. (2004). Intrinsically Motivated Reinforcement Learning. Proceedings of the Advances in Neural Information Processing Systems.

[68] MacKay D.J. (1992). Information-Based Objective Functions for Active Data Selection. Neural Comput..

[69] Cohn D., Ghahramani Z., Jordan M. (1994). Active Learning with Statistical Models. Proceedings of the Advances in Neural Information Processing Systems.

[70] Schmidhuber J. (2010). Formal Theory of Creativity, Fun, and Intrinsic Motivation (1990–2010). IEEE Trans. Auton. Ment. Dev..

[71] Kolter J.Z., Ng A.Y. Near-Bayesian Exploration in Polynomial Time. Proceedings of the 26th Annual International Conference on Machine Learning.

[72] Sun Y., Gomez F., Schmidhuber J., Schmidhuber J., Thórisson K.R., Looks M. (2011). Planning to Be Surprised: Optimal Bayesian Exploration in Dynamic Environments. Proceedings of the 4th International Conference on Artificial General Intelligence.

[73] Mohamed S., Jimenez Rezende D. (2015). Variational Information Maximisation for Intrinsically Motivated Reinforcement Learning. Proceedings of the Advances in Neural Information Processing Systems.

[74] Houthooft R., Chen X., Chen X., Duan Y., Schulman J., De Turck F., Abbeel P. (2016). VIME: Variational Information Maximizing Exploration. Proceedings of the Advances in Neural Information Processing Systems.

[75] Kim H., Kim J., Jeong Y., Levine S., Song H.O. EMI: Exploration with Mutual Information. Proceedings of the 36th International Conference on Machine Learning, PMLR.

[76] Eysenbach B., Gupta A., Ibarz J., Levine S. (2018). Diversity Is All You Need: Learning Skills without a Reward Function. arXiv.

[77] Sekar R., Rybkin O., Daniilidis K., Abbeel P., Hafner D., Pathak D. Planning to Explore via Self-Supervised World Models. Proceedings of the 37th International Conference on Machine Learning, PMLR.

[78] Zintgraf L., Schulze S., Lu C., Feng L., Igl M., Shiarlis K., Gal Y., Hofmann K., Whiteson S. (2021). VariBAD: Variational Bayes-Adaptive Deep RL via Meta-Learning. J. Mach. Learn. Res..

[79] Russo D., Van Roy B. (2018). Learning to Optimize via Information-Directed Sampling. Oper. Res..

[80] Lu X., Roy B.V., Dwaracherla V., Ibrahimi M., Osband I., Wen Z. (2023). Reinforcement Learning, Bit by Bit. Found. Trends^®^ Mach. Learn..

[81] Arumugam D., Roy B.V. Deciding What to Learn: A Rate-Distortion Approach. Proceedings of the 38th International Conference on Machine Learning, PMLR.

[82] Sukhija B., Coros S., Krause A., Abbeel P., Sferrazza C. MaxInfoRL: Boosting Exploration in Reinforcement Learning through Information Gain Maximization. Proceedings of the Thirteenth International Conference on Learning Representations.

[83] Lewis R.L., Howes A., Singh S. (2014). Computational Rationality: Linking Mechanism and Behavior through Bounded Utility Maximization. Top. Cogn. Sci..

[84] Griffiths T.L., Lieder F., Goodman N.D. (2015). Rational Use of Cognitive Resources: Levels of Analysis between the Computational and the Algorithmic. Top. Cogn. Sci..

[85] Lieder F., Griffiths T.L. (2019). Resource-Rational Analysis: Understanding Human Cognition as the Optimal Use of Limited Computational Resources. Behav. Brain Sci..

[86] Bhui R., Lai L., Gershman S.J. (2021). Resource-Rational Decision Making. Curr. Opin. Behav. Sci..

[87] Prystawski B., Mohnert F., Tošić M., Lieder F. (2022). Resource-Rational Models of Human Goal Pursuit. Top. Cogn. Sci..

[88] Zhu J.Q., Griffiths T.L. (2025). Computation-Limited Bayesian Updating: A Resource-Rational Analysis of Approximate Bayesian Inference. Psychol. Rev..

[89] Simon H.A. (1955). A Behavioral Model of Rational Choice. Q. J. Econ..

[90] de Clippel G., Rozen K. (2024). Bounded Rationality in Choice Theory: A Survey. J. Econ. Lit..

[91] Ortega P.A., Braun D.A. (2013). Thermodynamics as a Theory of Decision-Making with Information-Processing Costs. Proc. R. Soc. A Math. Phys. Eng. Sci..

[92] Ortega P.A., Braun D.A., Dyer J., Kim K.E., Tishby N. (2015). Information-Theoretic Bounded Rationality. arXiv.

[93] Gottwald S., Braun D.A. (2019). Bounded Rational Decision-Making from Elementary Computations That Reduce Uncertainty. Entropy.

[94] Harré M.S. (2021). Information Theory for Agents in Artificial Intelligence, Psychology, and Economics. Entropy.

[95] Marzen S. (2025). Resource-Rational Reinforcement Learning and Sensorimotor Causal States, and Resource-Rational Maximiners. arXiv.

[96] Shenhav A., Botvinick M.M., Cohen J.D. (2013). The Expected Value of Control: An Integrative Theory of Anterior Cingulate Cortex Function. Neuron.

[97] Kurzban R., Duckworth A., Kable J.W., Myers J. (2013). An Opportunity Cost Model of Subjective Effort and Task Performance. Behav. Brain Sci..

[98] Westbrook A., Braver T.S. (2015). Cognitive Effort: A Neuroeconomic Approach. Cogn. Affect. Behav. Neurosci..

[99] Gershman S.J., Horvitz E.J., Tenenbaum J.B. (2015). Computational Rationality: A Converging Paradigm for Intelligence in Brains, Minds, and Machines. Science.

[100] Shenhav A., Cohen J.D., Botvinick M.M. (2016). Dorsal Anterior Cingulate Cortex and the Value of Control. Nat. Neurosci..

[101] Russell S., Wefald E. (1991). Principles of Metareasoning. Artif. Intell..

[102] Russell S.J., Subramanian D. (1994). Provably Bounded-Optimal Agents. J. Artif. Intell. Res..

[103] Lieder F., Plunkett D., Hamrick J.B., Russell S.J., Hay N.J., Griffiths T.L. (2014). Algorithm Selection by Rational Metareasoning as a Model of Human Strategy Selection. Proceedings of the Advances in Neural Information Processing Systems.

[104] Lieder F., Griffiths T.L. (2017). Strategy Selection as Rational Metareasoning. Psychol. Rev..

[105] Sims C.A. (2003). Implications of Rational Inattention. J. Monet. Econ..

[106] Matějka F., McKay A. (2015). Rational Inattention to Discrete Choices: A New Foundation for the Multinomial Logit Model. Am. Econ. Rev..

[107] Maćkowiak B., Matějka F., Wiederholt M. (2023). Rational Inattention: A Review. J. Econ. Lit..

[108] Jaynes E.T. (1957). Information Theory and Statistical Mechanics. Phys. Rev..

[109] Jaynes E.T. (1957). Information Theory and Statistical Mechanics. II. Phys. Rev..

[110] Rosenkrantz R.D. (2012). E. T. Jaynes: Papers on Probability, Statistics and Statistical Physics.

[111] Cover T.M. (2006). Elements of Information Theory.

[112] Shore J., Johnson R. (1980). Axiomatic Derivation of the Principle of Maximum Entropy and the Principle of Minimum Cross-Entropy. IEEE Trans. Inf. Theory.

[113] Kullback S. (1997). Information Theory and Statistics.

[114] Csiszar I. (1975). I-Divergence Geometry of Probability Distributions and Minimization Problems. Ann. Probab..

[115] Kesavan H., Kapur J. (1989). The Generalized Maximum Entropy Principle. IEEE Trans. Syst. Man, Cybern..

[116] Jaynes E.T. (1980). The Minimum Entropy Production Principle. Annu. Rev. Phys. Chem..

[117] Prigogine I. (1978). Time, Structure, and Fluctuations. Science.

[118] Ortega P.A., Braun D.A. (2010). A Minimum Relative Entropy Principle for Learning and Acting. J. Artif. Intell. Res..

[119] Hafner D., Ortega P.A., Ba J., Parr T., Friston K., Heess N. (2022). Action and Perception as Divergence Minimization. arXiv.

[120] Boyd S.P. (2004). Convex Optimization.

[121] Ziebart B.D., Maas A., Bagnell J.A., Dey A.K. Maximum Entropy Inverse Reinforcement Learning. Proceedings of the 23rd National Conference on Artificial Intelligence.

[122] McKelvey R.D., Palfrey T.R. (1995). Quantal Response Equilibria for Normal Form Games. Games Econ. Behav..

[123] Goeree J.K., Holt C.A., Palfrey T.R. (2016). Quantal Response Equilibrium: A Stochastic Theory of Games.

[124] Scharfenaker E., Foley D.K. (2017). Quantal Response Statistical Equilibrium in Economic Interactions: Theory and Estimation. Entropy.

[125] Scharfenaker E. (2020). Implications of Quantal Response Statistical Equilibrium. J. Econ. Dyn. Control..

[126] Wolpert D.H., Harré M., Olbrich E., Bertschinger N., Jost J. (2012). Hysteresis Effects of Changing the Parameters of Noncooperative Games. Phys. Rev. E.

[127] Harré M.S., Atkinson S.R., Hossain L. (2013). Simple Nonlinear Systems and Navigating Catastrophes. Eur. Phys. J. B.

[128] Harris A., McCallum S., Harré M.S. (2023). On the Smooth Unfolding of Bifurcations in Quantal-Response Equilibria. Games Econ. Behav..

[129] Thom R. (1977). Structural Stability, Catastrophe Theory, and Applied Mathematics. SIAM Rev..

[130] Monderer D., Shapley L.S. (1996). Potential Games. Games Econ. Behav..

[131] Leonardos S., Piliouras G. (2022). Exploration-Exploitation in Multi-Agent Learning: Catastrophe Theory Meets Game Theory. Artif. Intell..

[132] Fox R., Mcaleer S.M., Overman W., Panageas I. Independent Natural Policy Gradient Always Converges in Markov Potential Games. Proceedings of the 25th International Conference on Artificial Intelligence and Statistics, PMLR.

[133] Barkley Rosser J. (2007). The Rise and Fall of Catastrophe Theory Applications in Economics: Was the Baby Thrown out with the Bathwater?. J. Econ. Dyn. Control..

[134] Harré M.S. (2022). Entropy, Economics, and Criticality. Entropy.

[135] Evans B.P., Prokopenko M. (2021). A Maximum Entropy Model of Bounded Rational Decision-Making with Prior Beliefs and Market Feedback. Entropy.

[136] Savin C., Tkačik G. (2017). Maximum Entropy Models as a Tool for Building Precise Neural Controls. Curr. Opin. Neurobiol..

[137] Schneidman E., Berry M.J., Segev R., Bialek W. (2006). Weak Pairwise Correlations Imply Strongly Correlated Network States in a Neural Population. Nature.

[138] Tkacik G., Marre O., Mora T., Amodei D., Berry M.J., Bialek W. (2013). The Simplest Maximum Entropy Model for Collective Behavior in a Neural Network. J. Stat. Mech. Theory Exp..

[139] Shannon C.E. (1948). A Mathematical Theory of Communication. Bell Syst. Tech. J..

[140] Shannon C.E. (1993). Coding Theorems for a Discrete Source with a Fidelity Criterion. Claude E. Shannon: Collected Papers; Institute of Radio Engineers, International Convention Record.

[141] Berger T. (1971). Rate Distortion Theory: A Mathematical Basis for Data Compression.

[142] Still S. (2009). Information-Theoretic Approach to Interactive Learning. Europhys. Lett..

[143] Tishby N., Polani D., Cutsuridis V., Hussain A., Taylor J.G. (2011). Information Theory of Decisions and Actions. Perception-Action Cycle: Models, Architectures, and Hardware.

[144] Blahut R. (1972). Computation of Channel Capacity and Rate-Distortion Functions. IEEE Trans. Inf. Theory.

[145] Arimoto S. (1972). An Algorithm for Computing the Capacity of Arbitrary Discrete Memoryless Channels. IEEE Trans. Inf. Theory.

[146] Poole B., Ozair S., Oord A.V.D., Alemi A., Tucker G. On Variational Bounds of Mutual Information. Proceedings of the 36th International Conference on Machine Learning, PMLR.

[147] Maisto D., Friston K., Pezzulo G. (2019). Caching Mechanisms for Habit Formation in Active Inference. Neurocomputing.

[148] Varona M.d.L., Buckley C.L., Millidge B. (2024). Exploring Action-Centric Representations Through the Lens of Rate-Distortion Theory. arXiv.

[149] Tishby N., Pereira F.C., Bialek W. (2000). The Information Bottleneck Method. arXiv.

[150] Creutzig F., Globerson A., Tishby N. (2009). Past-Future Information Bottleneck in Dynamical Systems. Phys. Rev. E.

[151] Alemi A.A. Variational Predictive Information Bottleneck. Proceedings of the 2nd Symposium on Advances in Approximate Bayesian Inference, PMLR.

[152] Stengel R.F. (1994). Optimal Control and Estimation.

[153] Bertsekas D., Shreve S.E. (1996). Stochastic Optimal Control: The Discrete-Time Case.

[154] Levine S. (2018). Reinforcement Learning and Control as Probabilistic Inference: Tutorial and Review. arXiv.

[155] Imohiosen A., Watson J., Peters J., Verbelen T., Lanillos P., Buckley C.L., De Boom C. (2020). Active Inference or Control as Inference? A Unifying View. Proceedings of the 2020 International Workshop on Active Inference.

[156] Bellman R., Kalaba R. (1959). A Mathematical Theory of Adaptive Control Processes. Proc. Natl. Acad. Sci. USA.

[157] Puterman M.L. (2014). Markov Decision Processes: Discrete Stochastic Dynamic Programming.

[158] Bellman R. (1953). An Introduction to the Theory of Dynamic Programming.

[159] Blackwell D. (1965). Discounted Dynamic Programming. Ann. Math. Stat..

[160] Watkins C.J.C.H., Dayan P. (1992). Q-Learning. Mach. Learn..

[161] Attias H. Planning by Probabilistic Inference. Proceedings of the International Workshop on Artificial Intelligence and Statistics, PMLR.

[162] Todorov E. (2006). Linearly-Solvable Markov Decision Problems. Proceedings of the Advances in Neural Information Processing Systems.

[163] Todorov E. General Duality between Optimal Control and Estimation. Proceedings of the 2008 47th IEEE Conference on Decision and Control.

[164] Toussaint M. Robot Trajectory Optimization Using Approximate Inference. Proceedings of the 26th Annual International Conference on Machine Learning.

[165] Theodorou E.A. (2015). Nonlinear Stochastic Control and Information Theoretic Dualities: Connections, Interdependencies and Thermodynamic Interpretations. Entropy.

[166] Song Z., Parr R., Carin L. Revisiting the Softmax Bellman Operator: New Benefits and New Perspective. Proceedings of the 36th International Conference on Machine Learning, PMLR.

[167] Kalman R.E. (1960). A New Approach to Linear Filtering and Prediction Problems. J. Basic Eng..

[168] Basar T. (2001). Contributions to the Theory of Optimal Control. Control Theory: Twenty-Five Seminal Papers (1932–1981).

[169] Sutton R.S., Barto A.G. (2018). Reinforcement Learning: An Introduction.

[170] Kochenderfer M.J., Wheeler T.A., Wray K.H. (2022). Algorithms for Decision Making.

[171] Ziebart B.D. (2010). Modeling Purposeful Adaptive Behavior with the Principle of Maximum Causal Entropy. Ph.D. Thesis.

[172] Maccheroni F., Marinacci M., Rustichini A. (2006). Ambiguity Aversion, Robustness, and the Variational Representation of Preferences. Econometrica.

[173] Hansen L.P., Sargent T.J. (2011). Robustness.

[174] Eysenbach B., Levine S. Maximum Entropy RL (Provably) Solves Some Robust RL Problems. Proceedings of the International Conference on Learning Representations.

[175] Ramírez-Ruiz J., Grytskyy D., Mastrogiuseppe C., Habib Y., Moreno-Bote R. (2024). Complex Behavior from Intrinsic Motivation to Occupy Future Action-State Path Space. Nat. Commun..

[176] Schultz W., Dayan P., Montague P.R. (1997). A Neural Substrate of Prediction and Reward. Science.

[177] Dayan P., Niv Y. (2008). Reinforcement Learning: The Good, The Bad and The Ugly. Curr. Opin. Neurobiol..

[178] Glimcher P.W. (2011). Understanding Dopamine and Reinforcement Learning: The Dopamine Reward Prediction Error Hypothesis. Proc. Natl. Acad. Sci. USA.

[179] Peters J., Mülling K., Altün Y. Relative Entropy Policy Search. Proceedings of the Twenty-Fourth AAAI Conference on Artificial Intelligence.

[180] Vieillard N., Kozuno T., Scherrer B., Pietquin O., Munos R., Geist M. (2020). Leverage the Average: An Analysis of KL Regularization in Reinforcement Learning. Proceedings of the Advances in Neural Information Processing Systems.

[181] Haarnoja T., Tang H., Abbeel P., Levine S. Reinforcement Learning with Deep Energy-Based Policies. Proceedings of the 34th International Conference on Machine Learning, PMLR.

[182] Haarnoja T., Zhou A., Abbeel P., Levine S. Soft Actor-Critic: Off-Policy Maximum Entropy Deep Reinforcement Learning with a Stochastic Actor. Proceedings of the 35th International Conference on Machine Learning, PMLR.

[183] Schulman J., Chen X., Abbeel P. (2018). Equivalence Between Policy Gradients and Soft Q-Learning. arXiv.

[184] Beck A., Teboulle M. (2003). Mirror Descent and Nonlinear Projected Subgradient Methods for Convex Optimization. Oper. Res. Lett..

[185] Hutter M. (2005). Universal Artificial Intelligence: Sequential Decisions Based on Algorithmic Probability.

[186] Rathmanner S., Hutter M. (2011). A Philosophical Treatise of Universal Induction. Entropy.

[187] Ross S., Chaib-Draa B., Pineau J. (2007). Bayes-Adaptive POMDPs. Proceedings of the Advances in Neural Information Processing Systems.

[188] Ghavamzadeh M., Mannor S., Pineau J., Tamar A. (2015). Bayesian Reinforcement Learning: A Survey. Found. Trends^®^ Mach. Learn..

[189] Arumugam D., Ho M.K., Goodman N.D., Van Roy B. (2024). Bayesian Reinforcement Learning with Limited Cognitive Load. Open Mind.

[190] Hafner D., Lillicrap T., Fischer I., Villegas R., Ha D., Lee H., Davidson J. Learning Latent Dynamics for Planning from Pixels. Proceedings of the 36th International Conference on Machine Learning, PMLR.

[191] Hafner D., Pasukonis J., Ba J., Lillicrap T. (2024). Mastering Diverse Domains through World Models. arXiv.

[192] Janner M., Fu J., Zhang M., Levine S. (2019). When to Trust Your Model: Model-Based Policy Optimization. Proceedings of the Advances in Neural Information Processing Systems.

[193] Ha D., Schmidhuber J. (2018). Recurrent World Models Facilitate Policy Evolution. Proceedings of the Advances in Neural Information Processing Systems.

[194] Matsuo Y., LeCun Y., Sahani M., Precup D., Silver D., Sugiyama M., Uchibe E., Morimoto J. (2022). Deep Learning, Reinforcement Learning, and World Models. Neural Netw..

[195] Richens J., Everitt T., Abel D. General Agents Need World Models. Proceedings of the Forty-Second International Conference on Machine Learning.

[196] Moerland T.M., Broekens J., Plaat A., Jonker C.M. (2023). Model-Based Reinforcement Learning: A Survey. Found. Trends^®^ Mach. Learn..

[197] Ha D., Schmidhuber J. (2018). World Models. arXiv.

[198] Schrittwieser J., Antonoglou I., Hubert T., Simonyan K., Sifre L., Schmitt S., Guez A., Lockhart E., Hassabis D., Graepel T. (2020). Mastering Atari, Go, Chess and Shogi by Planning with a Learned Model. Nature.

[199] Shalizi C.R. (2009). Dynamics of Bayesian Updating with Dependent Data and Misspecified Models. Electron. J. Stat..

[200] Crutchfield J.P., Feldman D.P. (2003). Regularities Unseen, Randomness Observed: Levels of Entropy Convergence. Chaos.

[201] Hoffman M.D., Johnson M.J. ELBO Surgery: Yet Another Way to Carve up the Variational Evidence Lower Bound. Proceedings of the NeurIPS Workshop on Advances in Approximate Bayesian Inference.

[202] Friston K., Parr T., Heins C., Da Costa L., Salvatori T., Tschantz A., Koudahl M., Van de Maele T., Buckley C., Verbelen T. (2025). Gradient-Free De Novo Learning. Entropy.

[203] Friston K.J., Da Costa L., Tschantz A., Kiefer A., Salvatori T., Neacsu V., Koudahl M., Heins C., Sajid N., Markovic D. (2024). Supervised Structure Learning. Biol. Psychol..

[204] Friston K., Da Costa L., Hafner D., Hesp C., Parr T. (2021). Sophisticated Inference. Neural Comput..

[205] Kaufmann R., Gupta P., Taylor J. (2021). An Active Inference Model of Collective Intelligence. Entropy.

[206] Albarracin M., Pitliya R.J., St. Clere Smithe T., Friedman D.A., Friston K., Ramstead M.J.D. (2024). Shared Protentions in Multi-Agent Active Inference. Entropy.

[207] Hyland D., Gavenčiak T., Costa L.D., Heins C., Kovarik V., Gutierrez J., Wooldridge M.J., Kulveit J. Free-Energy Equilibria: Toward a Theory of Interactions Between Boundedly-Rational Agents. Proceedings of the ICML 2024 Workshop on Models of Human Feedback for AI Alignment.

[208] Ruiz-Serra J., Sweeney P., Harré M.S., Vorobeychik Y., Das S., Nowé A. (2025). Factorised Active Inference for Strategic Multi-Agent Interactions. Proceedings of the 24th International Conference on Autonomous Agents and Multiagent Systems (AAMAS 2025).

[209] Paul A., Sajid N., Da Costa L., Razi A. (2024). On Efficient Computation in Active Inference. Expert Syst. Appl..

[210] Maisto D., Gregoretti F., Friston K.J., Pezzulo G. (2025). Active Inference Tree Search in Large POMDPs. Neurocomputing.

[211] Fountas Z., Sajid N., Mediano P., Friston K. (2020). Deep Active Inference Agents Using Monte-Carlo Methods. Proceedings of the Advances in Neural Information Processing Systems.

[212] Champion T., Grześ M., Bowman H. (2022). Branching Time Active Inference with Bayesian Filtering. Neural Comput..

[213] Champion T., Grześ M., Bowman H. (2024). Multimodal and Multifactor Branching Time Active Inference. Neural Comput..

[214] Mazzaglia P., Verbelen T., Dhoedt B. Contrastive Active Inference. Proceedings of the Advances in Neural Information Processing Systems 2021.

[215] Nuijten W.W.L., Lukashchuk M., van de Laar T., de Vries B. (2025). A Message Passing Realization of Expected Free Energy Minimization. arXiv.

[216] Papadimitriou C.H., Tsitsiklis J.N. (1987). The Complexity of Markov Decision Processes. Math. Oper. Res..

[217] Madani O., Hanks S., Condon A. (2003). On the Undecidability of Probabilistic Planning and Related Stochastic Optimization Problems. Artif. Intell..

